# Trifluoromethyl-substituted 3,5-bis(arylidene)-4-piperidones as potential anti-hepatoma and anti-inflammation agents by inhibiting NF-кB activation

**DOI:** 10.1080/14756366.2021.1953996

**Published:** 2021-07-20

**Authors:** Wei Cong, Yue Sun, Yi-Fan Sun, Wei-Bin Yan, Yu-Long Zhang, Zhong-Fei Gao, Chun-Hua Wang, Gui-Ge Hou, Jia-Jing Zhang

**Affiliations:** School of Pharmacy, the Key Laboratory of Prescription Effect and Clinical Evaluation of State Administration of Traditional Chinese Medicine of China, Binzhou Medical University, Yantai, P. R. China

**Keywords:** 3,5-Bis(arylidene)-4-piperidones, anti-hepatoma, anti-inflammation, NF-κB inhibitor, trifluoromethyl

## Abstract

Some methoxy-, hydroxyl-, pyridyl-, or fluoro-substituted 3,5-bis(arylidene)-4-piperidones (BAPs) could reduce inflammation and promote hepatoma cell apoptosis by inhibiting activation of NF-κB, especially after introduction of trifluoromethyl. Herein, a series of trifluoromethyl-substituted BAPs (**4-30**) were synthesised and the biological activities were evaluated. We successfully found the most potential **16**, which contains three trifluoromethyl substituents and exhibits the best anti-tumour and anti-inflammatory activities. Preliminary mechanism research revealed that **16** could promote HepG2 cell apoptosis in a dose-dependent manner by down-regulating the expression of Bcl-2 and up-regulating the expression of Bax, C-caspase-3. Meanwhile, **16** inhibited activation of NF-κB by directly inhibiting the phosphorylation of p65 and IκB*α* induced by LPS, together with indirectly inhibiting MAPK pathway, thereby exhibiting both anti-hepatoma and anti-inflammatory activities. Molecular docking confirmed that **16** could bind to the active sites of Bcl-2, p65, and p38 reasonably. The above results suggested that **16** has enormous potential to be developed as a multifunctional agent for the clinical treatment of liver cancers and inflammatory diseases.

## Introduction

Liver cancer is one of the most common diseases in the world and causes more than 830,180 deaths each year, which account for about 8.3% of the total annual cancer deaths. Unfortunately, the number of cancer deaths worldwide is increasing, and liver cancer is considered as the third leading cause of cancer deaths in 2020^1^. In China, the people suffering from liver cancer and dying from this disease account for about 20% of the global-related toll^2^. Hepatocellular carcinoma (HCC) accounts for about 80% of the related type of primary liver cancer^3^. Numerous studies have shown that the nuclear factor-kappa B (NF-κB) is one of the key signalling pathway molecules linking HCC and chronic inflammation^4–10^.

The mammalian NF-κB is a protein complex that contains p65 (RelA), p52, p50, RelB, and c-Rel factors^11^. Under normal physiological conditions, NF-κB is in an inactive state and exists as a dimer with an inhibiting protein IκB. IκB kinase (IKK) can be easily activated under the stimulation of many proinflammatory cytokines and other factors such as tumour necrosis factor-α (TNF-α), and nitric oxide (NO). After IκB is phosphorylated by IKK, p62 is released from IκB. Then, NF-κB dimer will be released, transferred to the nucleus and mediate the target genes activation^12^^,^^13^.

Inflammatory factors, such as TNF-α, IL-1, IL-6, IL-17, and COX-2, can activate NF-κB^14^^,^^15^. Studies have shown that HCC cell apoptosis will be prevented if NF-κB is activated. It will trigger the tumour cells to continue to proliferate and increase drug resistance^16^^,^^17^. By inhibiting the excessive activation of NF-κB, it can inhibit the production of inflammation, thus causing HCC apoptosis and reducing the production of liver cancer^18^^,^^19^. Until now, there is no ideal drug targeting NF-κB for the treatment of HCC. Therefore, it is of great importance to study the mechanism of action and synthesis methods of such drugs for the development of NF-κB inhibitors.

**Figure 1.**
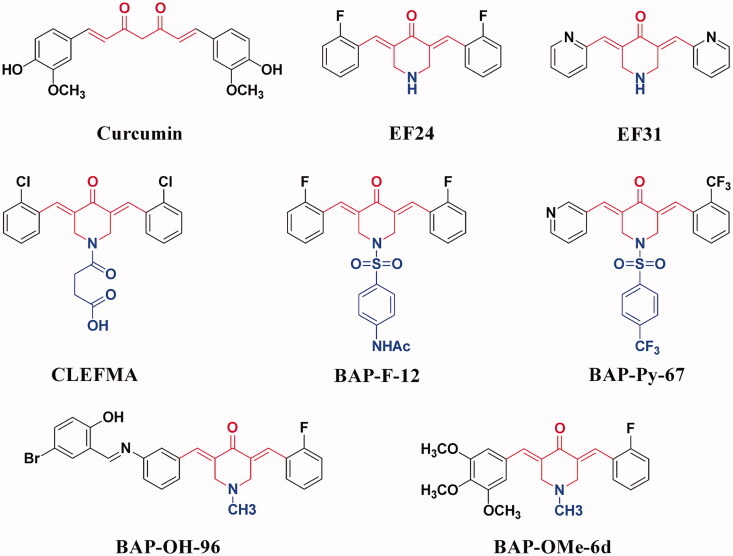
The structures of curcumin and some reported BAPs.

Among the numerous studies that directly or indirectly target NF-κB pathway, many compounds synthesised or derived from the structural modification of natural products show remarkable activities, such as oxazines^20^, biscoumarins^21^, and curcumins^22^. Curcumin (Figure 1) has a variety of biological activities, but its clinical application is limited due to its low bioavailability and poor stability^22^. Therefore, based on the structure and literature reports of curcumin, the structural optimisation and synthesis of curcumin analogues have attracted people's attention. Therein, (*3E,5E*)-3,5-bis(arylene)-4-piperidone (BAP) is a typical example. It has good biological activity, which can achieve anti-inflammatory and anti-tumour effects by inhibiting the activation of NF-κB^23–26^. In our group, we designed and synthesised a series of methoxy-, hydroxyl-, pyridyl-, or fluoro-substituted BAPs (Figure 1), which could significantly inhibit the activation of NF-κB signalling pathway by blocking the phosphorylation of IκBα, p65 and the nuclear translocation of NF-κB, thus exhibiting both anti-tumour and anti-inflammatory activities^27–35^. Compared with other lead compounds that directly or indirectly act on NF-κB pathway, such as natural curcumin, the BAPs we designed and synthesised are easy to synthesise, modify and have stable chemical properties.

In our previous study, we found that the anti-tumour and anti-inflammatory activities of BAPs were superior after the introduction of the trifluoromethyl group in the aromatic ring, which may be attributed to the introduction of fluorine atoms to further affect the biological activity. As a strong electron-withdrawing substitution, the trifluoromethyl group can alter the electronic effects of target compounds, and its lipophilicity can effectively improve the membrane permeability of target compounds. Furthermore, they can form multiple hydrogen or halogen bonds with the target protein to improve biological activities^35^. Therefore, we proposed to introduce more trifluoromethyl substitutions on both sides of BAPs to further optimise the biological activities. Our efforts led to the discovery of trifluoromethyl-substituted BAPs, which might become potential anti-hepatoma and anti-inflammation agents by inhibiting the activation of NF-κB.

## Experimental

### Materials and methods

4-Piperidinone hydrate hydrochloride, 2-(trifluoromethyl)benzaldehyde, 3-(trifluoromethyl)benzaldehyde, 4-(trifluoromethyl)benzaldehyde, and several benzenesulphonyl chlorides are purchased from Shanghai Bide Pharmatech Ltd. (China) and do not require further purification. ^1^H NMR data were collected using a Bruker Avance 400 or 600 MHz. ^13 ^C NMR data were collected at 100 MHz on a Bruker Avance 400 MHz spectrometer or 150 MHz on a Bruker Avance 600 MHz spectrometer. Chemical shifts were reported in δ relative to TMS. Infra-red (IR) spectra were obtained in the 400–4000 cm^q−1^ range using a Perkin-Elmer Frontier Mid-IR FTIR Spectrometer. The HRMS-ESI data were obtained on a Finnigan-MAT-95 mass spectrometer. All melting points were measured on a digital melting point apparatus and were uncorrected.

### Preparation of BAPs 4–30

In a 50-ml beaker, piperidone hydrochloride **1** (1.51 g, 0.01 mol) and trifluoromethyl-substituted benzaldehyde **2** (3.48 g, 0.02 mol) were dissolved in 25 ml of acetic acid. After aerated with dry HCl gas for 45 min, the mixture was stirring for 2 days at room temperature. After the addition of 25 ml acetone, the precipitate (denoted as **3**) was filtered and used directly for the next reaction without further treatment. **3** (0.45 g, 1.0 mmol) and substituted benzenesulphonyl chloride (1.0 mmol) were dissolved in 50 ml of DCM. After adding four drops of pyridine, the mixture was stirred at room temperature overnight. The reaction was monitored by TLC. After the reaction was completed, the solution was washed three times with dilute hydrochloric acid, dried over anhydrous Na_2_SO_4_ and concentrated under reduced pressure to obtain the yellow solid, which was recrystallized with DCM/MeOH (1:1, v/v) to get yellow crystals of BAPs **4–30**. Compounds **23**, **24**, **26** and **29** were characterised in a literature^32^. Others were confirmed by NMR, FTIR, and HRMS (Supporting Information).

### Single-crystal structure determination of 5

Suitable single crystals of BAP **5** were prepared by recrystallization via solvent evaporation in DCM and MeOH solution under room temperature. It measured at 100 K with Mo Kα radiation (*λ* = 0.71073 Å) using a Rigaku OD SuperNova Dual source diffractometer with an AtlasS2 detector. Absorption was corrected with multi-scan methods. The structure was refined in SHELXL-2017/1^36^. Crystal data of **5**: C_28_H_21_F_6_NO_3_S, *M* = 565.52, triclinic, space group *P-1*, clear light yellow plate, *a* = 12.2154(7) Å, *b* = 15.0290(10) Å*, c* = 15.1516(10) Å, *α = 83.506(5)*°, *β* = 82.037(5)°, *γ = 66.058(6)*°, *V* = 2512.8(3) Å^3^, Z = 4, *D*c = 1.495 g·cm^−3^, *μ*(Mo-Kα) = 0.206 mm^−1^, *T* = 100(2) K. 23388 unique reflections [*R*_int_ = 0.0771]. Final *R*_1_ [with *I* > 2σ(I)] = 0.0603, *wR*_2_ (all data) = 0.1409. CCDC 1982277 (**5**) contains the supplementary crystallographic data for this paper. Copies of the data can be obtained free of charge on application to CCDC, 12 Union Road, Cambridge CB2 1EZ, UK (fax: (+44)1223-336-033; e-mail: deposit@ccdc.cam.ac.uk).

### In vitro anti-tumour activity testing of BAPs (4–30) with MTT method

To measure anti-tumour activity, two Human hepatoma cell lines (HepG2, SMMC-7721) and one human normal heptical cell line (LO2) were screened by BAPs (**4–30**) using a modified MTT assay (Dojindo Laboratories, Tokyo, Japan). The HepG2, SMMC-7721, and LO2 cell lines were maintained at 37 °C, 5% humidified CO_2_, and 95% atmosphere. Dulbecco's Modified Eagle (DMEM) medium containing 10% foetal bovine serum (FBS) was used for culturing the HepG2 cell line; meanwhile, Roswell Park Memorial Institute (RPMI) 1640 medium containing 10% FBS was used for culturing other cell lines (SMMC-7721, LO2). The HepG2, SMMC-7721 and LO2 cells were seeded in a 96-well plate in 200 μL of medium per well at a density of approximately 8 × 10^3^ cells/well and cultured for 24 h, followed by incubation in an incubator of indicated compounds with successive concentrations (10, 5, 2.5, 1.25, 0.625, 0.3125 μg/mL) for 24 h. In the control group, cells were only cultured with culture media. After the media were removed, 20 μL of MTT (5 mg/mL) was added, then cells were incubated for 4 h at 37 °C. Next, removed the media with MTT and added 150 μL of DMSO to dissolve the dark-blue formazan crystals. The optical density (OD) value of each well was measured on a multi-well plate reader (TECAN, Männedorf, Switzerland) at 570 nm. GraphPad Prism 5 software was used to calculate their IC_50_ values. Every IC_50_ value was the average of three replicates. Doxorubicin (DOX) and curcumin were used as positive controls. The concentrations of DOX used were 5, 2.5, 1.25, 0.625, 0.3125, 0.15625 μg/mL. The concentrations of Curcumin used were 100, 50, 25, 12.5, 6.25, 3.125, 1, 0.5 and 0.1 μg/mL. Results are the average of three replicates and shown in Table 1.

**Table 1.** Cytotoxicity of BAPs (**4–30**), curcumin, and DOX.

Compound	R_1_	R_2_	HepG2(μM)	SI^a^	SMMC-7721(μM)	SI^a^	LO2(μM)
**4**	2-CF_3_	-H	9.2 ± 0.2	1.8	8.2 ± 0.6	2.0	16.4 ± 0.2
**5**	2-CF_3_	-CH_3_	17.5 ± 0.1	2.1	19.7 ± 0.1	1.8	36.2 ± 2.3
**6**	2-CF_3_	-NO_3_	4.3 ± 0.2	3.7	10.4 ± 0.2	1.5	15.7 ± 1.3
**7**	2-CF_3_	-CF_3_	2.1 ± 0.1	8.2	2.6 ± 0.2	6.6	17.2 ± 0.2
**8**	2-CF_3_	-F	4.0 ± 0.2	3.5	8.3 ± 0.2	1.7	13.8 ± 1.1
**9**	2-CF_3_	-Cl	11.0 ± 0.3	2.8	10.4 ± 0.3	3.0	30.9 ± 0.3
**10**	2-CF_3_	-Br	14.7 ± 0.1	1.2	4.7 ± 0.1	3.6	17.0 ± 0.4
**11**	2-CF_3_	-CN	9.0 ± 0.2	1.4	12.0 ± 0.2	1.1	13.0 ± 0.1
**12**	2-CF_3_	-NHAc	12.9 ± 0.1	1.2	10.9 ± 0.4	1.4	15.6 ± 1.3
**13**	3-CF_3_	-H	3.5 ± 0.1	3.1	5.7 ± 0.1	1.8	10.1 ± 0.4
**14**	3-CF_3_	-CH_3_	11.1 ± 0.2	1.4	5.6 ± 0.1	2.8	15.9 ± 0.1
**15**	3-CF_3_	-NO_3_	2.7 ± 0.1	4.7	5.0 ± 0.1	2.5	12.6 ± 0.1
**16**	3-CF_3_	-CF_3_	0.6 ± 0.2	29.7	1.2 ± 0.2	14.8	17.8 ± 0.5
**17**	3-CF_3_	-F	3.4 ± 0.2	5.7	4.8 ± 0.3	4.1	19.5 ± 0.1
**18**	3-CF_3_	-Cl	5.2 ± 0.1	2.7	13.9 ± 0.1	1.0	14.1 ± 0.1
**19**	3-CF_3_	-Br	6.5 ± 0.2	1.7	7.6 ± 0.4	1.4	11.0 ± 0.2
**20**	3-CF_3_	-CN	0.9 ± 0.1	19.3	4.3 ± 0.2	4.0	17.4 ± 0.1
**21**	3-CF_3_	-NHAc	1.3 ± 0.1	11.1	5.1 ± 0.1	2.8	14.4 ± 0.1
**22**	4-CF_3_	-H	1.8 ± 0.2	8.0	8.5 ± 0.2	2.1	18.2 ± 0.2
**23**	4-CF_3_	-CH_3_	1.0 ± 0.3	13.1	0.8 ± 0.2	16.4	13.1 ± 0.3
**24**	4-CF_3_	-NO_3_	1.2 ± 0.3	12.0	3.2 ± 0.3	4.5	14.4 ± 0.1
**25**	4-CF_3_	-CF_3_	0.9 ± 0.2	20.3	1.3 ± 0.3	14.1	18.3 ± 0.6
**26**	4-CF_3_	-F	3.1 ± 0.2	5.1	3.1 ± 0.1	5.1	15.7 ± 0.2
**27**	4-CF_3_	-Cl	1.4 ± 0.2	11.3	1.8 ± 0.2	8.8	15.8 ± 0.4
**28**	4-CF_3_	-Br	1.7 ± 0.2	8.4	1.9 ± 0.2	7.5	14.2 ± 0.3
**29**	4-CF_3_	-CN	1.7 ± 0.1	11.4	1.3 ± 0.3	16.5	21.5 ± 1.5
**30**	4-CF_3_	-NHAc	1.8 ± 0.2	7.9	3.1 ± 0.2	4.6	14.2 ± 1.1
DOX	–	–	1.3 ± 0.3	6.8	1.7 ± 0.2	5.2	8.9 ± 0.5
Curcumin	–	–	15.3 ± 2.5	1.9	19.5 ± 1.3	1.5	29.1 ± 2.1

^a^The letters SI refer to the selectivity index which is the quotient of the IC_50_ values for normal and malignant cells.

### Anti-inflammatory testing of BAPs (4–30)

We tested by inhibiting the secretion of TNF-α and IL-6 to better evaluated the anti-inflammatory effects of BAPs. In our pre-experiments, all BAPs (**4–30**) did not show obvious toxicity to mouse RAW264.7 macrophagocyte at the concentration of 5.0 μM. Under the stimulation of LPS (1.0 μg/mL) with RAW264.7 cells, the release of TNF-α and IL-6 was detected. Besides, after treated with BAPs (5.0 μM) and LPS (1.0 μg/mL), the release of TNF-*α* and IL-6 was measured with an ELISA kit (eBioScience, San Diego, California), respectively. In general terms, pyrrolidine dithiocarbamate (PDTC, 30 μM) or BAP (5.0 μM) was used to pretreat cells for 2 h, respectively. Then, LPS (1.0 μg/mL) was added and cells were incubated for another 22 h. Finally, the culture media were centrifuged at 1000 rpm for 10 min, and the expression levels of TNF-*α* and IL-6 were determined by ELISA. Similarly, the inhibition rates are the means of three replicates and are as shown in Table 2.

**Table 2.** Anti-inflammatory activity of BAPs (**4–30**) against inflammatory response in LPS-stimulated RAW264.7 cell.

No.	Survival rate (%)	Inhibition rate (%)	
	BAPs (5 μM)	BAPs (5 μM) + LPS (1 μg/mL)	
	RAW264.7 cell	TNF-α	IL-6
**4**	82.2 ± 2.0	62.1 ± 4.7	70.7 ± 1.3
**5**	93.3 ± 1.7	46.2 ± 1.0	49.6 ± 1.1
**6**	85.7 ± 3.1	61.0 ± 2.7	68.2 ± 3.0
**7**	87.3 ± 1.9	69.9 ± 1.1	71.2 ± 1.3
**8**	82.1 ± 1.9	46.8 ± 4.2	51.3 ± 3.3
**9**	92.8 ± 4.2	47.1 ± 6.4	38.2 ± 2.5
**10**	83.8 ± 2.9	59.2 ± 2.8	67.8 ± 3.2
**11**	84.2 ± 2.0	58.9 ± 3.2	59.1 ± 5.2
**12**	86.4 ± 0.7	67.2 ± 2.4	62.8 ± 3.0
**13**	82.5 ± 1.3	56.3 ± 2.1	48.9 ± 2.9
**14**	89.2 ± 1.8	60.2 ± 1.2	66.3 ± 2.0
**15**	81.9 ± 1.2	62.1 ± 0.7	60.8 ± 4.2
**16**	88.6 ± 3.7	83.2 ± 2.4	79.2 ± 1.4
**17**	85.1 ± 2.0	65.6 ± 1.2	67.6 ± 2.2
**18**	85.7 ± 4.2	50.6 ± 3.2	46.2 ± 2.1
**19**	81.9 ± 0.6	49.2 ± 3.7	56.3 ± 1.3
**20**	83.7 ± 1.3	75.2 ± 2.7	77.7 ± 1.5
**21**	90.2 ± 3.6	78.5 ± 4.2	71.6 ± 5.3
**22**	89.4 ± 7.8	65.2 ± 1.3	68.3 ± 1.3
**23**	89.2 ± 3.0	78.1 ± 3.2	69.2 ± 6.3
**24**	89.0 ± 1.6	65.0 ± 1.8	57.3 ± 2.1
**25**	87.3 ± 4.2	81.3 ± 1.7	75.9 ± 1.9
**26**	87.6 ± 1.7	66.3 ± 5.3	62.8 ± 3.0
**27**	86.4 ± 4.8	69.9 ± 2.7	67.3 ± 2.2
**28**	84.0 ± 3.6	70.4 ± 2.0	70.1 ± 1.2
**29**	83.4 ± 3.3	72.5 ± 1.4	74.2 ± 3.2
**30**	92.0 ± 1.3	68.3 ± 3.1	70.1 ± 1.2
LPS	–	0	0
PDTC(30 μM)	–	68.2 ± 2.0	59.1 ± 1.6

### Apoptosis assay

HepG2 cells were selected and plated at a density of approximately 2 × 10^5^ cells/well in a 12-well plate. After treatment with BAP **16** (1.0, 2.0, and 4.0 μM) and DMSO for 24 h, cells were harvested, washed twice with pre-chilled PBS, and suspended in 1 × binding buffer at a concentration of 1 × 10^5^ cells/mL. According to the manufacturer’s instruction, 100 μL of such medium was then mixed with 5 μL of Annexin V-FITC and 5 μL of propidium iodide (BD Biosciences, San Jose, CA, USA). The mixture was vortexed gently, then incubated in the dark at room temperature for 15 min. Apoptosis analysis was measured by flow cytometry (BD FACS Calibur).

### Western blot assay

Primary antibodies (anti-Bax, Abcam, ab32503; anti-Bcl-2, Abcam, ab194583; anti-Cleaved caspase-3, Abcam, ab2302; anti-p65, Cell Signalling Technology, 8242S; anti-*p*-p65, Cell Signalling Technology, 3033S; anti-IκB*α*, Cell Signalling Technoligy, 4812S; anti-*p*-IκB*α*, Cell Signalling Technology, 2859S; anti-p38, Cell Signalling Technology, 8690S; anti-*p*-p38, Cell Signalling Technology, 4511S; anti-JNK, Cell Signalling Technology, 9258 T; anti-*p*-JNK, Santa Cruz Biotechnology, sc-6254; anti-GAPDH, Abcam, ab181602), and goat anti-rabbit IgG/HRP secondary antibodies (Cell Signalling Technology, 98164 s) were purchased and used according to the manufacturer’s instructions. HepG2 cell lines were incubated with different doses of BAP **16** (1.0, 2.0, 4.0 μM) for 24 h (Bax, Cleaved caspase-3, Bcl-2). RAW264.7 cells were incubated with BAP **16** for 1 h, then treated with LPS (1.0 μg/mL) for 30 min. Afterward cell extraction was performed and extracts were used for Western blot analysis. Next, 30 μg protein of cell lysates were separated by 12% SDS-PAGE gel. The proteins were then transferred onto nitrocellulose membranes. The membranes were probed with either anti-Bax, anti-Cleaved caspase-3, anti-Bcl-2, anti-IκB*α*, anti-phospho-IκB*α*, anti-p65, anti-phospho-p65, anti-JNK, anti-phospho-JNK, anti-p38, anti-phospho-p38 or anti-GAPDH (1:1000 dilution for all primary antibodies) at 4 °C overnight. Lastly, the membrane was incubated for 2 h at room temperature with goat anti-rabbit IgG/HRP secondary antibodies (1:1000 dilution), and the ECL Western blot detection system (ChemiDoc^TM^XRS, Bio-Rad) was used to detect the expression of each protein in the membrane. By using Quantity One software (Bio-Rad), western blotting results were quantified from more than three separate experiments.

### Molecular docking

The PDB files for the crystal structures of Bcl-2, NF-κB/p65, and p38 were obtained using the protein data bank codes 1YSW, 1MY5, and 4FA2, respectively. The molecular docking procedure was performed under the C-DOCKER protocol of Accelry’s Discovery Studio 2017R2 software. For ligand preparation, the structure of BAP **16** was constructed using ChemDraw Professional 17.0 software, saved in SDF file format and minimised using Accelry’s Discovery Studio 2017R2 software. The protein structures were cleaned and inspected for errors, hydrogens were added, and the water molecules were deleted. For Bcl-2 protein as a receptor, the centroid of the binding site was defined based on the ligand in the cocrystal structure. Then removed the original ligand and placed the molecule of BAP **16** in the sphere position to carry out molecular docking. Subsequently, p65 and P38 proteins were defined as receptors and docked in a similar process. For energy minimisation, the CHARMM force field was utilised within Accelry’s Discovery Studio 2017R2 software. Finally, types of interactions between the docked proteins and BAP **16** were analysed.

## Results and discussion

### Synthesis and structural characterisation

**Scheme 1.**
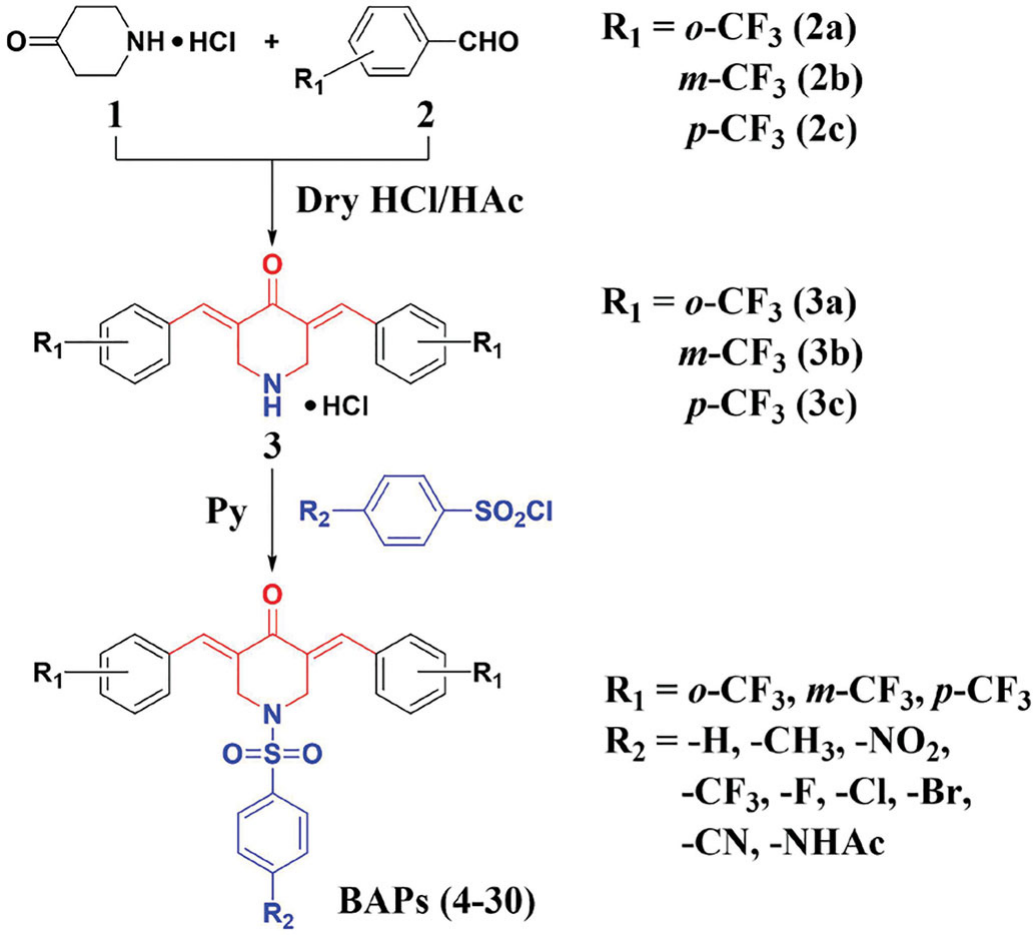
The synthetic routes of BAPs (**4–30**).

The synthetic routes to BAPs (**4–30**) are shown in Scheme 1. Preparation of BAPs includes Claisen-Schmidt condensation and *N*-sulphonylation reaction. Firstly, using 4-piperidinone hydrochloride (**1**) and *o*-, *m*- or *p*-trifluoromethyl benzaldehyde (**2a**, **2 b** or **2c**) as starting materials, the reaction of Claisen-Schmidt condensation was catalysed by dry hydrogen chloride gas to generate *o*-, *m*-, or *p*-trifluoromethyl-substituted intermediates (BAP-H, **3a**, **3 b** or **3c)**, in the form of hydrochloride. Secondly, title compounds *N*-phenylsulphonyl-BAPs (BAPs **4–30**) were synthesised by *N*-sulphonylation reaction. This protocol was carried out through BAP-H and *para*-substituted benzenesulphonyl chloride (including hydrogen, methyl, nitro, trifluoromethyl, halogen, cyano, and acetylamino) under pyridine catalysis. It should be noted that pyridine was a key reagent in the above process, which acted as a catalyst as well as an acid-binding agent to neutralise the hydrogen chloride from both BAP-H hydrochloride and the *N*-sulphonylation reaction. Finally, after the above two steps, we were pleased to obtain the title compounds BAPs (**4–30**) in moderate to good yields (76%∼90%). Then, their chemical structures were confirmed by ^1^H NMR, ^13 ^C NMR, FTIR, and HRMS.

Due to the similar skeleton (3*E*,5*E*)-3,5-bis(arylidene)-4-piperidone, the spectral characteristics of the title compounds BAPs (**4–30**) have some common features. Take BAP **4** as an example. In ^1^H NMR spectra, the chemical shift in 7.81 ppm appears as a singlet attributed to the two protons of the symmetrical *α*,*β*-unsaturated ketone pharmacophore. Likewise, ^1^H NMR signals of two methylene groups of piperidone overlap into one single peak with the chemical shift in 4.52 ppm. In ^13 ^C NMR spectra, the characteristic peak with a chemical shift in 183.66 ppm should correspond to the carbonyl carbon atom of piperidone, and the band in 46.80 ppm attributes to the two methylene carbon atoms of piperidone. In conclusion, the above signals in ^1^H NMR and ^13 ^C NMR prove the existence of (3*E*,5*E*)-3,5-bis(arylidene)-4-piperidone pharmacophore. In FTIR spectra, the strong characteristic absorption band of the carbonyl group can be found in 1676 cm^−1^. The distinct bands of carbon-carbon double bonds in *α*,*β*-unsaturated ketone and aromatic ring are shown in around 1584 cm^−1^. Besides, the strong bands at around 1159 cm^−1^ are attributed to the group of sulphamide (-SO_2_N-). Moreover, HRMS results demonstrate that the measured molecular weights of BAPs are basically consistent with the calculated values. These results further confirm the correctness of their structures.

**Figure 2.**
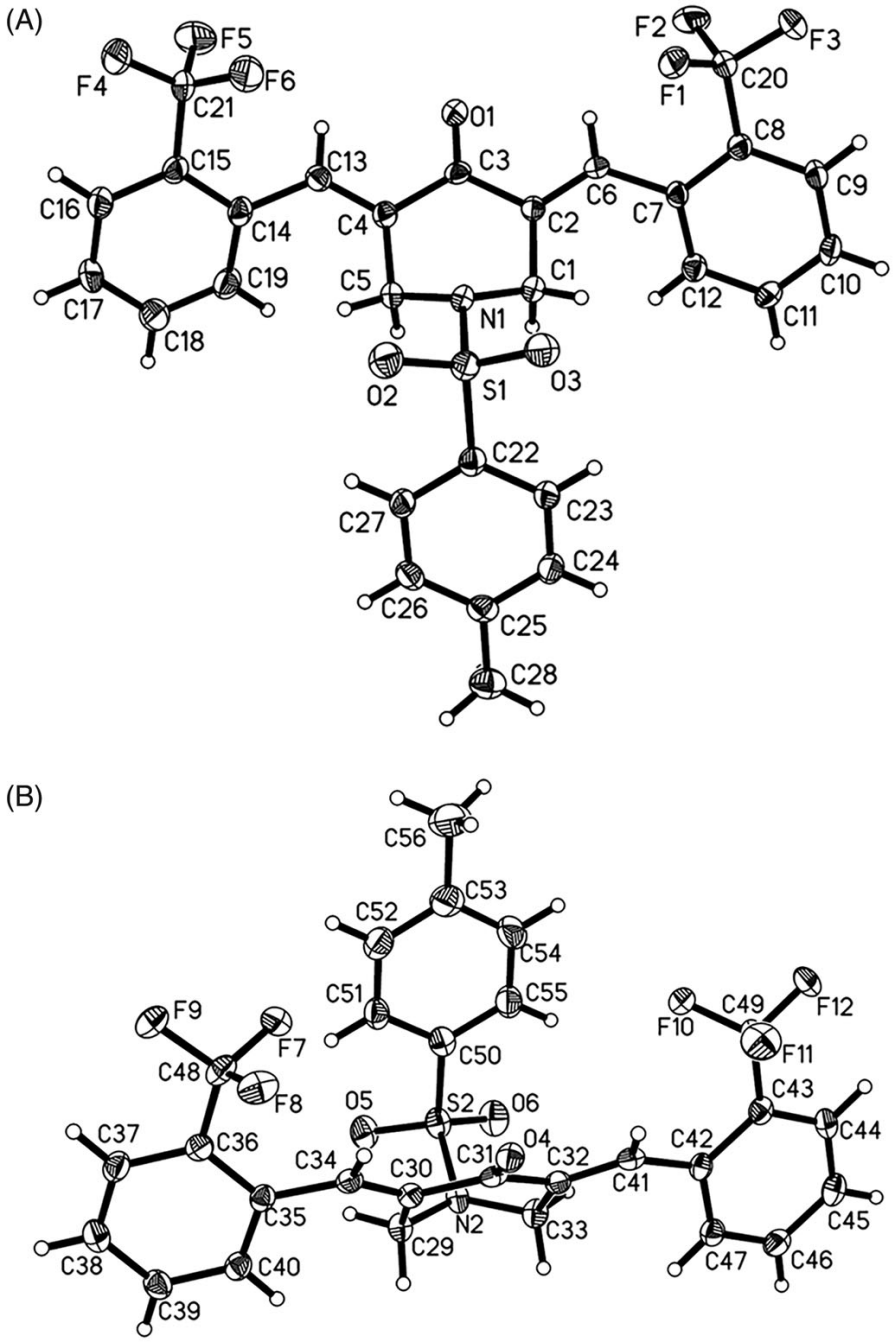
The ORTEP figure of BAP **5** (Two molecules A and B with displacement ellipsoids of 50% probability).

Single crystals of BAP **5** were prepared under ambient conditions, with crystallisation obtained via solvent evaporation in a dichloromethane/methanol solution. Single-crystal structure analysis revealed that that there are two molecules in the asymmetric unit of **5** (Figure 2). Bond lengths and angles are all in the expected ranges. In both molecules, arylidene on both sides of central piperidone adopt the *E* stereochemistry of the olefinic double bonds. In one molecule, the 2-(trifluoromethyl)benzylidene substituents are going to stretch in the direction of the carbonyl group of central piperidone, while *N*-phenylsulfonyl group extends in the opposite direction. The dihedral angles between 2-(trifluoromethyl) benzylidene and central piperidone are 51.4(3)° and 47.2(4)°, and the dihedral angle between *p*-methylphenyl group and central piperidone are 44.1(4)°. Whereas, in another molecule, *N*-phenylsulphonyl group extends in the same direction of the carbonyl group of central piperidone. The dihedral angles between *p*-methylphenyl group and piperidone ring are 36.4(3)°. Polytropic configuration could provide more possibilities for better biological activity, while the peripheric heteroatoms (Such as F, N, O, S) can act as H-bonding acceptors for bioactive molecules with the aim of creating more potent bioactivities^30^.

### Cytotoxicity, anti-inflammatory activity and structure–activity relationship (SAR) analysis of BAPs (4–30)

To investigate the *in vitro* cytotoxicity of BAPs (**4–30**), two Human hepatoma cell lines (HepG2, SMMC-7721) and one human normal heptical cell line (LO2) were screened by a modified MTT assay. DOX and curcumin were selected as positive controls. The selectivity index (SI) was the quotient of the IC_50_ values for normal and malignant cells, and the results were summarised in Table 1. The title compounds BAPs **4-12** bearing the same 2-CF_3_ substituent of R_1_ on both benzene rings of (3*E*,5*E*)-3,5-bis(arylidene)-4-piperidone, exhibited discrepant anti-tumour activities due to the different substituents of R_2_ on the *para*-position of the benzenesulphonyl part. Herein, we focussed on the investigation of the substituent effect of R_2_. With a hydrogen group of R_2_, BAP **4** demonstrated moderate anti-tumour activity (ca. 9.2 μM against HepG2; ca. 8.2 μM against SMMC-7721), which was superior to curcumin (ca. 15.3 μM against HepG2; ca. 19.5 μM against SMMC-7721), but weaker than DOX (ca. 1.3 μM against HepG2; ca. 1.7 μM against SMMC-7721). However, anti-tumour activities decreased when changed the hydrogen group into a methyl group (BAP **4** *vs* **5**). In consideration of the electron-donating or non-polar property of methyl, we tried to introduce some electron-withdrawing or polar substituents at the *para*-position of the benzenesulphonyl part, such as nitro, trifluoromethyl, halogen, cyano, or acetylamino (BAPs **6-12**). Among them, we were delighted to find that BAP **7** bearing a trifluoromethyl group of R_2_, exhibited good anti-tumour activities (ca. 2.1 μM against HepG2; ca. 2.6 μM against SMMC-7721), which was comparable with DOX. However, replacing trifluoromethyl with other electron-withdrawing or polar substituents gave no further improved results. Encouraged by this result, then we investigated the anti-tumour activities of BAPs with a 3-CF_3_ substituent of R_1_ on both benzene rings of (3*E*,5*E*)-3,5-bis(arylidene)-4-piperidone skeleton. Compared with BAPs bearing the 2-CF_3_ substituent of R_1_ (BAPs **4-12**), similar SAR regularities could be found in BAPs bearing the 3-CF_3_ substituent of R_1_ (BAPs **13-21**). Among BAPs **13-21**, the most noticeable compound was BAP **16**, bearing a trifluoromethyl group of R_2_, which exhibited potent anti-tumour activities (ca. 0.6 μM against HepG2; ca. 1.2 μM against SMMC-7721). It should be noted that, although the anti-tumour activities were superior to DOX, BAP **16** displayed relatively weaker cytotoxicity against normal heptical cells (ca. 17.8 μM against LO2), which could be demonstrated by the SI values (ca. 29.7 for HepG2; ca. 14.8 for SMMC-7721). Beyond that, compounds bearing cyano or acetylamino group of R_2_ (BAPs **20** and **21**), displayed relatively good anti-tumour activities against HepG2 (0.9 μM and 1.3 μM, respectively), which was comparable with DOX. Very interestingly, the anti-tumour activities dramatically improved when BAPs bearing 4-CF_3_ substituent of R_1_ (BAPs **22-30**). For example, BAP **23** with a methyl group of R_2_, exhibited more potent anti-tumour activities (ca. 1.0 μM against HepG2; ca. 0.8 μM against SMMC-7721) than that with a hydrogen group of R_2_ (BAP **22**: ca. 1.8 μM against HepG2; ca. 8.5 μM against SMMC-7721), and this result was quite different from previous similar compounds bearing a 2-CF_3_ or 3-CF_3_ substituent of R_1_ (BAP **5** *vs* **4** and BAP **14** *vs* **13**). In effect, attributed to the 4-CF_3_ substituent of R_1_, this series of BAPs (**22-30**) universally demonstrated good anti-tumour activities, with the IC_50_ values range from 0.9 μM to 1.8 μM against HepG2 and 0.8 μM to 8.5 μM against SMMC-7721. Furthermore, considering the selectivity to tumour cells and normal cells, BAPs **23**, **25**, and **29** bearing a 4-CF_3_ substituent of R_1_ were as potent as BAP **16** and worthy of further study.

To further verify the anti-tumour activity, one human non-small cell lung cancer cell line (A549), one human ovarian cancer cell line (A2780), and one human normal heptical cell line (HHL-5) were screened by BAPs (**4–30**) using a modified MTT assay (Supporting Information). DOX and curcumin were selected as positive controls. To our delight, most of the BAPs showed good anti-tumour activity. At the same time, it also showed a similar rule with the previous experimental results, that was, the anti-tumour activity of the BAPs bearing a 3-CF_3_ or 4-CF_3_ substituent of R_1_ were significantly better than that of the 2-CF_3_ substitution. Especially when R_1_ was 4-CF_3_ substituted, R_2_ was bromo- or cyano-substituted compounds (BAPs **28** and **29**) showed better anti-tumour activity and selectivity (BAP **29**: ca. 0.081 μM against A549; ca. 0.18 μM against A2780; SI > 90) than the positive drug DOX (ca. 0.29 μM against A549; ca. 0.19 μM against A2780; SI > 40). These results indicated that BAPs not only had potential therapeutic value for liver cancer, but also some of them were expected to be further developed as candidate drugs for the treatment of lung, ovarian and other cancers.

Based on the above results, preliminary SAR of anti-tumour activities could be summarised. Firstly, the substituent position of the R_1_ group on both benzene rings of (3*E*,5*E*)-3,5-bis(arylidene)-4-piperidone skeleton played a key role in anti-tumour effects, and 3-CF_3_ or 4-CF_3_ substituent significantly outperformed 2-CF_3_ substituent. Secondly, the substituent property of the R_2_ group on *para*-position of the benzenesulphonyl part also distinctly affected the anti-tumour activities, and electron-withdrawing or polar substituents such as the trifluoromethyl group could effectively improve the bioactivities.

Lipopolysaccharide (LPS) is a key constituent of Gram-negative bacterial endotoxin, which is used to induce an inflammatory response in corresponding inflammatory cells^37^. TNF-*α* and IL-6 are two kinds of representative pro-inflammatory factors, which produced by LPS-stimulated RAW264.7 macrophage^38^. In our pre-experiments, the toxicity against RAW264.7 macrophagocyte for all title compounds was tested by the MTT assay, and the results were listed in Table 2. The survival rates were above 80% for all BAPs (**4-30**) at the concentration of 5.0 μM. Thus, the above results suggested that BAPs (**4-30**) did not show obvious toxicity to RAW264.7 cells.

In the following study, we detected TNF-α and IL-6 secretion from LPS-pre-treated RAW264.7 macrophagocyte with BAPs (**4-30**) by ELISA method and took pyrrolidine dithiocarbamate (PDTC, 30 μM) as a positive control to determine the anti-inflammatory activities. For BAPs with a 2-CF_3_ substituent of R_1_ (BAPs **4–12**), BAP **7** exhibited higher inhibition rates (ca. 69.9% against TNF-α; ca. 71.2% against IL-6) than the positive control PDTC (ca. 68.2% against TNF-α; ca. 59.1% against IL-6), which bearing a trifluoromethyl group of R_2_. Among the 3-CF_3_ substituent series (BAPs **13–21**), several BAPs (**16**, **20**, and **21**) displayed more effective inhibition of inflammatory cytokines than that of PDTC. Based on the above results, polar groups such as trifluoromethyl (**16**), cyano (**20**), or acetylamino (**21**) substituted on the *para*-position of the benzenesulphonyl part might be beneficial to the anti-inflammatory activities. Remarkably, BAP **16** demonstrated unsurpassed anti-inflammatory activities (ca. 83.2% against TNF-α; ca. 79.2% against IL-6), which was head and shoulders above the positive control PDTC. To our delight, almost all BAPs with a 4-CF_3_ substituent of R_1_ (BAPs **22–30**) were superior to PDTC. Especially, BAPs bearing the trifluoromethyl (**25**), bromo (**28**), or cyano (**29**) group of R_2_ on the *para*-position of the benzenesulphonyl part, displayed more than 70% inhibition rates against IL-6 and TNF-α cytokine release. These foregoing results indicated that six BAPs (**16**, **20**, **21**, **25**, **28**, and **29**) displayed desired bioactivities, and the SAR of anti-inflammatory activities showed similar regularities as that of anti-tumour activities. Firstly, the substituent position of the R_1_ group was crucial, 3-CF_3_ or 4-CF_3_ substituent was superior to the 2-CF_3_ substituent. Secondly, the substituent property of the R_2_ group also played an important role in anti-inflammatory activities, and electron-withdrawing or polar substituents such as trifluoromethyl, halogen, cyano, or acetylamino group were beneficial to improve anti-inflammatory activities.

Combined with cytotoxicity activities previously discussed, it is obvious to discover that BAPs (**16**, **25**, and **29**) substituted by electron-withdrawing groups (trifluoromethyl or cyano) of R_2_ possess better cytotoxicity against malignant cells, less toxicity to normal cells, and more significant anti-inflammatory activities. Specifically, the cytotoxicity against HCC cells followed the order: **16** (average, 0.9 μM) >**25** (average, 1.1 μM) >**29** (average, 1.5 μM), and the SI value followed the order: **16** (average, 22.3) >**25** (average, 17.2) >**29** (average, 14). Meanwhile, the inhibition rates against IL-6 and TNF-α cytokine release followed the order: **16** (average, 81.2%) > **25** (average, 78.6%) > **29** (average, 73.4%), and the survival rate of RAW264.7 cells followed the order: **16** (88.6%) > **25** (87.3%) > **29** (83.4%).

In the above experiments, BAP **16** showed good anti-tumour and anti-inflammatory activities. In order to verify its safety, a preliminary safety evaluation was carried out (Supporting Information). First, at the cellular level, the modified MTT method was used to detect the cytotoxicity of BAP **16** on human embryonic lung fibroblast cell line (HFL1), human embryonic kidney cell line (HEK293), and human normal lung bronchial epithelial cell line (BEAS-2B). In brief, BAP **16** showed moderate cytotoxicity to this three human normal non-heptical cell lines (ca. 9.62 μM against HFL1; ca. 7.19 μM against HEK293; ca. 17.58 μM against BEAS-2B), which was similar to that of human normal heptical cell lines (ca. 17.08 μM against LO2). Second, in the acute toxicity studies, During the 14 days of intragastric administration (0, 300, 600 mg/kg/d), the food and water intakes of all three groups of mice were normal, the body weight increased slightly, and no obvious toxic reaction was observed. However, in the HE staining experiment, some tissues of mouse livers showed dose-related toxic changes, such as smaller hepatocyte volume, deeper nuclear staining, eosinophilic changes, and oedema. The above results showed that BAP **16** had low overall toxicity, but long-term high-dose use would produce toxic reactions.

**Figure 3.**
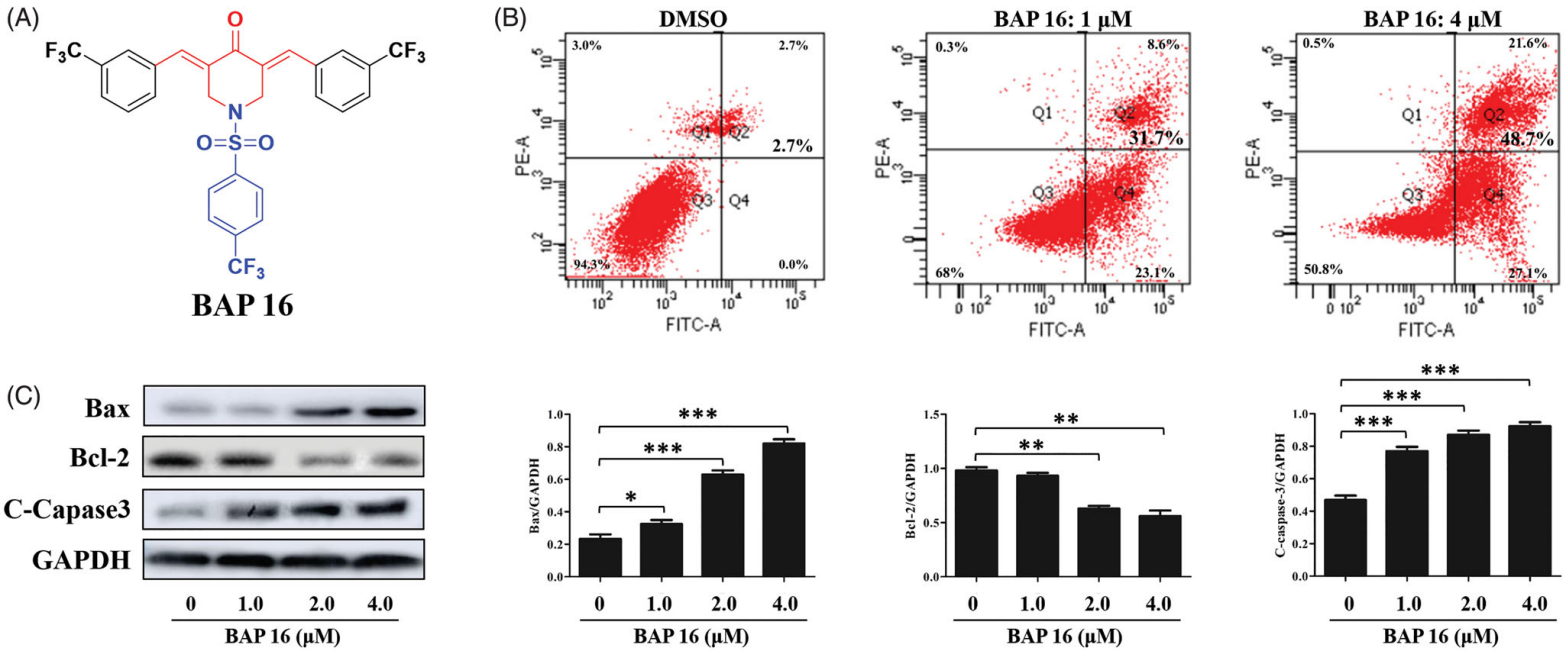
(A) The structure of BAP **16**. (B) Cell apoptotic of HepG2 by BAP **16** through flow cytometry. (C) The expression of Bax, Bcl-2, and C-Caspase-3 in HepG2 cells was determined by western blotting. Western blots were quantified from more than three separate experiments (*p < 0.05, **p < 0.01, ***p < 0.001 compared to DMSO group).

In conclusion, based on the above analysis, BAP **16** exhibited unsurpassed bioactivities and low overall toxicity which could be selected as a lead compound for further study. The structure of BAP **16** is shown in Figure 3(A).

### BAP 16-induced HepG2 cells apoptosis

The previous experiments disclosed that BAP **16** possessed prominent anti-tumour and anti-inflammatory activities. To investigate the underlying mechanism of repressed cell proliferation observed in the MTT assay, the experiments about cell apoptosis of HepG2 were carried out by flow cytometry. As shown in Figure 3(B), BAP **16** (1.0 μM) could induce 31.7% apoptosis for HepG2 cells. Moreover, with the concentration increasing to 4.0 μM, the rate of cell apoptosis could reach about 48.7%, which showed that BAP **16** induced HepG2 cell apoptosis in a dose-dependent manner.

At the protein level, several kinds of cell apoptotic proteins, such as anti-apoptotic protein Bcl-2 and pro-apoptotic proteins Bax and C-Caspase-3, play a key role in malignant tumour cell apoptosis^39^. Herein, the effects of BAP **16** on the expression levels of apoptosis-related proteins (Bax, Bcl-2, and C-Caspase-3) in HepG2 cells were investigated by Western blot. As shown in Figure 3(C), after treated by BAP **16** (1.0, 2.0, 4.0 μM), the expression of anti-apoptotic protein Bcl-2 was decreased in a dose-dependent manner. On the contrary, the expression of pro-apoptotic proteins Bax and C-Caspase-3 were significantly increased, also displayed a dose-dependent manner. In summary, BAP **16** could induce dose-dependent cell apoptosis of HepG2 through down-regulating the expression of Bcl-2 and up-regulating the expression of Bax and C-Caspase-3.

### BAP 16 inhibited the phosphorylation of IκB and p65 in RAW264.7 cells

**Figure 4.**
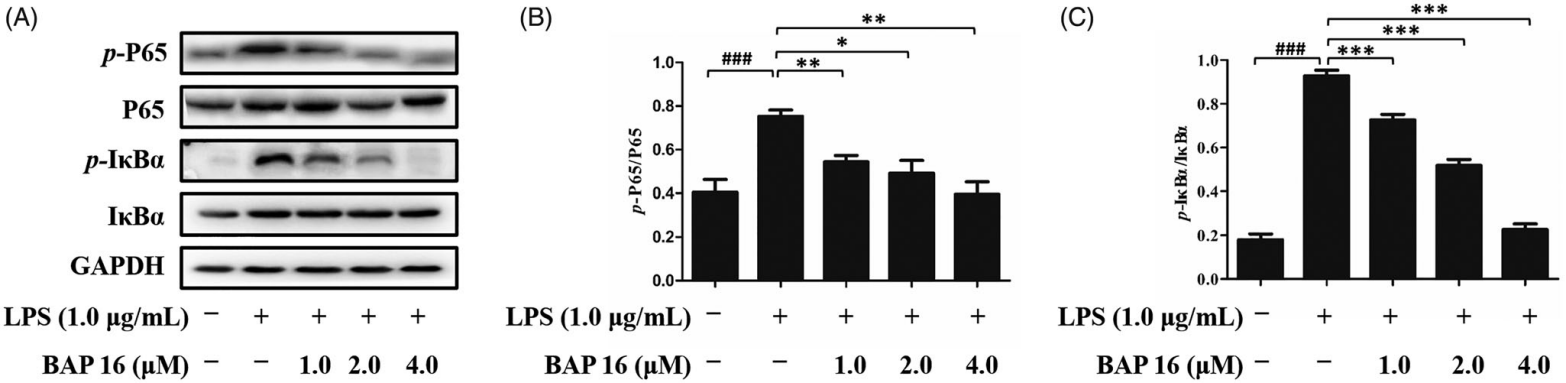
(A) Inhibitory effects of BAP **16** on LPS-stimulated phosphorylation of p65 (B), IκB*α* (C) in RAW264.7 cells, respectively. They were detected by Western blotting. GAPDH was used as a control. Data represent the mean ± SD of triplicate tests. *p < 0.05, **p < 0.01, ***p < 0.001 compared with LPS group. ^###^*p* < 0.001 compared with DMSO group.

NF-κB is an important signalling pathway molecule, which plays a key role at the crossroad of tumour proliferation and inflammatory response^7–10^. More than that, down-regulating of NF-κB and IκB phosphorylation could generate a significant inhibitory effect on cancer and inflammation^11^. In this case, it is essential to investigate whether BAP **16** exhibits anti-tumour and anti-inflammatory effects by inhibiting NF-κB activation. Based on this hypothesis, we conducted the following experiments by Western blotting. First, after stimulated by LPS, phosphorylation of p65 and IκB*α* occurred in RAW264.7 cells in 30 min (Figure 4(A)). Next, when treated with BAP **16** (1.0, 2.0, 4.0 μM), the expression levels of *p*-p65 and *p*-IκB*α* were significantly decreased in a dose-dependent manner (Figure 4(B,C)). In summary, these results revealed that BAP **16** could inhibit phosphorylation of p65 and IκB*α* and down-regulating the expression levels of *p*-p65 and *p*-IκB*α*, ultimately leading to the inhibition of NF-κB.

**Figure 5.**
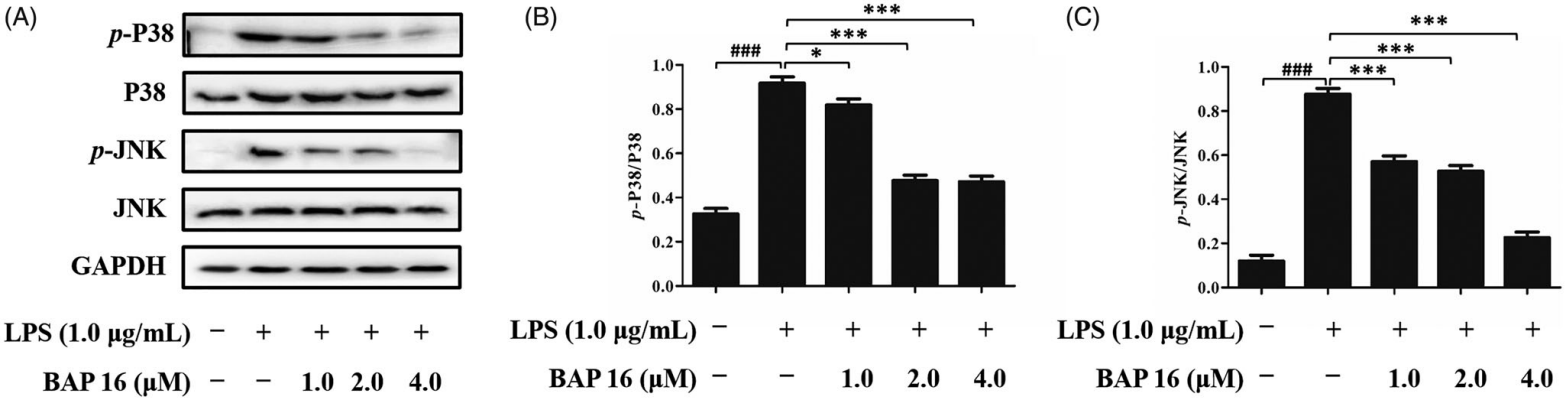
(A) Inhibitory effects of BAP **16** on LPS-induced MAPK signalling in RAW264.7 cells detected by Western blotting. Inhibitory effects of BAP **16** on LPS-induced phosphorylation of p38 (B) and JNK (C), respectively. GAPDH was used as a control. Data represent the mean ± SD of triplicate tests. *p < 0.05, **p < 0.01, ***p < 0.001 compared with LPS group. ^###^*p* < 0.001 compared with DMSO group.

Mitogen-activated protein kinase (MAPK) cascade pathway plays a key role in cell signal transduction, which involves in many physiological or pathological processes, such as inflammatory response, cellular proliferation, and apoptosis regulation^40^. Beyond that, c-jun *N*-terminal kinase (JNK) and p38 MAP kinase (p38) are two important members of the MAPK family. Based on the above reasons, it is indispensable to investigate the phosphorylation levels of JNK and p38 to determine whether BAP **16** displays anti-tumour and anti-inflammatory activities by affecting the MAPK signalling pathway. At first, RAW264.7 cells were stimulated by LPS, and the phosphorylation levels of two factors (p38 and JNK) significantly increased in 30 min (Figure 5(A)). However, once pre-treated with BAP **16** (1.0, 2.0, 4.0 μM), the expression levels of *p*-p38 and *p*-JNK were significantly decreased (Figure 5(B,C)). The above results suggested that BAP **16** could inhibit the phosphorylation of MAPKs, which might indirectly inhibit the activation of the NF-κB-signalling pathway.

In summary, based on the above results, we proposed a possible mechanism for the inhibition of the NF-κB-signalling pathway. BAP **16** could effectively inhibit the activation of the NF-κB pathway, mainly by down-regulating the phosphorylation levels of p65 and IκBα, and indirectly by suppressing the phosphorylation of MAPKs, ultimately leading to the reduction of inflammatory mediators.

### Molecular docking study

**Figure 6.**
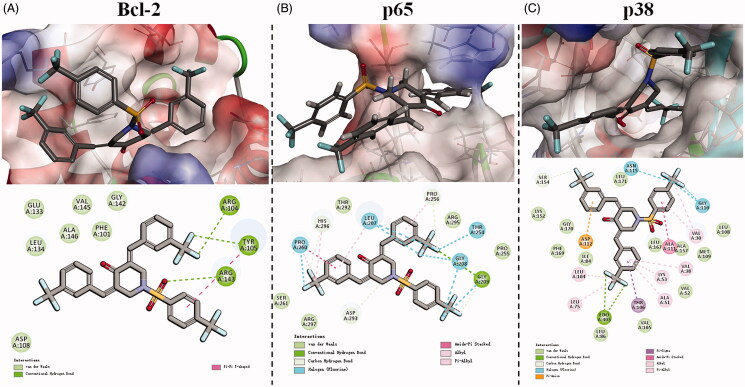
3D model and 2D model of the interaction between simulated BAP **16** and the active site of Bcl-2 (A), p65 (B), and p38 (C) proteins.

To further investigate the interaction modes between BAP **16** and Bcl-2, NF-κB/p65, and p38 proteins, we carried out molecular docking studies using the C-DOCKER module of Accelry’s Discovery Studio 2017R2 software. In the preparation stage, the chemical structure of BAP **16** was constructed and corrected, and the crystal structures of Bcl-2 (PDB: 1YSW), NF-κB/p65 (PDB: 1MY5), and p38 (PDB: 4FA2) proteins were optimized. The docking results were analysed and showed in Figure 6.

Figure 6(A) illustrates the hypothetical binding mode between BAP **16** and the active site of Bcl-2 protein. 4-Piperidone part of BAP **16** intercalates readily at the narrow groove on the surface of Bcl-2 protein. In detail, one of the 3-(trifluoromethyl) benzylidene moieties interacts with ARG 104 and TYR 105 through hydrogen bonds, while the other moiety inserts into a biggish hydrophobic pocket and interacts with some amino acid residues (such as ASP 108, GLU 133, LEU 134, and ALA 146) via van der Waals force and hydrophobic force. Furthermore, the 4-(trifluoromethyl) benzenesulphonyl part attaches to the surface of Bcl-2 protein by a hydrogen bond between the sulphonyl group and ARG 143, as well as π–π stacking interactions between the phenyl group and TYR 105.

The hypothetical binding mode between BAP **16** and the active site of NF-κB/p65 protein is shown in Figure 6(B). Notably, one of the 3-(trifluoromethyl) benzylidene moieties of BAP **16** crosses the channel of p65 protein, which is mainly stabilised by the halogen (fluorine) bond and amide-π stacking interactions with PRO 260. Beyond that, other forces like carbon-hydrogen bond, van der Waals, alkyl, and π-alkyl interactions with HIS 296, LEU 207, SER 261, and ARG 297 also contribute to stabilise the above process. It should be noted that the other two trifluoromethyl groups of BAP **16** also play a key role in binding to the p65 protein by hydrogen and halogen (fluorine) bonds, which may provide further explanation for the advantage of trifluoromethyl substitution.

The active site of p38 protein possesses a big pocket, which is completely different from Bcl-2 or NF-κB/p65 proteins. As shown in Figure 6(C), BAP **16** could exactly insert into this pocket and bind to the surrounding amino acid residues through relatively complex interactions. Among them, hydrogen and halogen (fluorine) bonds formed between trifluoromethyl groups and p38 protein are the most prominent interactions. In other words, the superiority of trifluoromethyl substitution could also be demonstrated by this result.

In summary, BAP **16** could reasonably bind to the active sites of Bcl-2, NF-κB/p65, and p38 proteins. These results could further verify the consistency of the aforementioned Western blot data with the anti-tumour and anti-inflammatory activities of BAP **16**.

## Conclusions

NF-κB family is an important pathway linking inflammation and cancer, which plays a key role in the development of hepatitis and liver cancer. Therefore, the NF-κB pathway has become an important target for the research of anti-inflammatory and anti-tumour drugs. In our previous study, we found that BAPs containing *N*-benzenesulphonyl group had stronger anti-inflammatory and anti-tumour activities, but lower toxicity to normal cells. At the same time, the introduction of trifluoromethyl groups into the benzene rings of BAP nucleus could also effectively improve the activity. In this experiment, 27 trifluoromethyl-substituted BAPs (**4–30**) were successfully designed and synthesised. Their pharmacological properties including anti-tumour and anti-inflammatory activities were primarily investigated at cell and protein levels. The structure-activity relationship (SAR) analysis of cytotoxicity and anti-inflammatory activities revealed that BAPs (**16**, **25**, and **29**) substituted by electron-withdrawing groups (trifluoromethyl or cyano) on *para*-position of the benzenesulphonyl part exhibited the better cytotoxicity against malignant cells, less toxicity to normal cells and more significant anti-inflammatory activities. Among them, BAP **16** bearing three trifluoromethyl substituents exhibited the most potent bioactivities.

Preliminary studies on the anti-tumour mechanism of BAP **16** disclosed that it could induce dose-dependent cell apoptosis of HepG2 cells through down-regulating the expression of Bcl-2 and up-regulating the expression of Bax and C-Caspase-3. Further studies indicated that BAP **16** could effectively inhibit the activation of the NF-κB pathway in RAW264.7 cells, mainly via down-regulating the phosphorylation levels of p65 and IκBα, and indirectly by suppression the phosphorylation of MAPKs, ultimately leading to the reduction of inflammatory mediators. Molecular docking analysis verified that BAP **16** could reasonably bind to the active site of Bcl-2, NF-κB/p65, and p38 proteins.

In conclusion, this study demonstrates that BAP **16**, as a representative of BAPs containing trifluoromethyl substitution, is a novel NF-κB inhibitor with desired biological activities and high research value. However, the current research on BAPs is still in the initial stage, and only part of the preclinical pharmacological studies has been completed. More than that, BAPs also have challenges such as insufficient druggability. In the future research work, we will continue to carry out in-depth evaluation of *in vitro* and *in vivo* pharmacological activities, as well as corresponding structural modifications to improve the druggability of BAPs, and finally develop them into multifunctional agents for the clinical treatment of inflammatory diseases and liver cancers.

## Supplementary Material

Supplemental Material
