# Supplementary material for: Trifluoromethyl-substituted 3,5-bis(arylidene)-4-piperidones as potential anti-hepatoma and anti-inflammation agents by inhibiting NF-кB activation
Source: J Enzyme Inhib Med Chem. 2021 Jul 20;36(1):1622–31. doi: 10.1080/14756366.2021.1953996 (PMC8297402; doi:10.1080/14756366.2021.1953996)
Supplement: Supplemental Material [file IENZ_A_1953996_SM3603.pdf]

Supporting Information

Trifluoromethyl-substituted 3,5-bis(arylidene)-4-piperidones as potential anti-hepatoma and anti-inflammation agents by inhibiting NF-κB activation

Wei Cong[1], Yue Sun[1], Yi-Fan Sun, Wei-Bin Yan, Yu-Long Zhang, Zhong-Fei Gao, Chun-Hua Wang*, Gui-Ge Hou*, Jia-Jing Zhang*

School of Pharmacy, the Key Laboratory of Prescription Effect and Clinical Evaluation of State Administration of Traditional Chinese Medicine of China, Binzhou Medical University, Yantai, 264003, P. R. China

Tel: +86-535-6913317; fax: +86-535-6913718;

***E-mail address: jiajing_z@163.com (J.-J. Zhang), chunhuawang508@126.com (C.H. Wang), guigehou@163.com (G.-G. Hou)*

[1]These authors contributed equally to this work.

Structural characterization

(3*E*,5*E*)-1-(phenylsulfonyl)-3,5-bis(2-(trifluoromethyl)benzylidene)piperidin-4-one (**4**)

Yellow powder, yield: 84%; m.p. 148~150°C. ^{1}H NMR (600 MHz, DMSO) δ 7.92 (d, *J* = 7.7 Hz, 2H), 7.83 (t, *J* = 7.8 Hz, 2H), 7.81 (s, 2H), 7.71 (m, 3H), 7.51 (m, 6H), 4.52 (s, 4H). ^{13}C NMR (150 MHz, DMSO) δ 183.66, 137.90, 134.07, 133.76, 133.36, 133.07 132.33, 131.25, 130.41, 129.96, 128.14 (q, *J* = 29.7 Hz), 127.37, 127.09 (q, *J* = 4.5 Hz), 124.42 (q, *J* = 273.0 Hz), 46.80. IR (cm^{-1}): 1676(s), 1584(s), 1487(s), 1349(s), 1290(s), 1239(s), 1159(s), 1115(m), 1100(s), 1063(s), 1033(s), 988(s), 823(m), 738(s). HRMS: calcd for $C_{27}H_{20}F_6NO_3S$ [M + H$^+$] 552.1068; found 552.1073.

(3*E*,5*E*)-1-tosyl-3,5-bis(2-(trifluoromethyl)benzylidene)piperidin-4-one (**5**)

Yellow powder, yield: 82%; m.p. 186~189°C. ^{1}H NMR (600 MHz, DMSO) δ 7.92 (d, *J* = 7.8 Hz, 2H), 7.80 (m, 4H), 7.70 (t, *J* = 7.4 Hz, 2H), 7.50 (d, *J* = 7.6 Hz, 2H), 7.39 (d, *J* = 7.8 Hz, 2H), 7.29 (d, *J* = 7.9 Hz, 2H), 4.46 (s, 4H), 2.37 (s, 3H). ^{13}C NMR (150 MHz, DMSO) δ 183.84, 144.51, 137.34, 134.82, 133.71, 133.35, 133.19, 132.36, 131.23, 130.39, 128.12 (q, *J* = 29.7 Hz), 127.46, 127.06 (q, *J* = 5.4 Hz), 124.41 (q, *J* = 272.3 Hz), 46.71, 20.94. IR (cm^{-1}): 2814(s), 1920(m), 1676(s), 1594(s), 1488(s), 1352(s), 1315(s), 1289(s), 1160(s), 1118(s), 1027(s), 992(s), 958(s), 839(s), 737(s). HRMS: calcd for $C_{28}H_{22}F_6NO_3S$ [M + H$^+$] 566.1225; found 566.1215.

(3*E*,5*E*)-1-((4-nitrophenyl)sulfonyl)-3,5-bis(2-(trifluoromethyl)benzylidene)piperidin-4-one (**6**)

Yellow powder, yield: 84%; m.p. 204~206°C. ^{1}H NMR (600 MHz, DMSO) δ 8.28 (d, *J* = 8.8 Hz, 2H), 7.92 (d, *J* = 7.8 Hz, 2H), 7.84 (m, 4H), 7.81 (d, *J* = 8.8 Hz, 2H), 7.72 (t, *J* = 7.7 Hz, 2H), 7.52 (d, *J* = 7.7 Hz, 2H), 4.56 (s, 4H). ^{13}C NMR (150 MHz, DMSO) δ 183.84, 150.46, 143.44, 134.05, 133.41, 132.80, 132.15, 131.22, 130.50, 129.10, 128.13 (q, *J* = 29.7 Hz), 127.1 (q, *J* = 5.5 Hz), 125.16, 124.4 (q, *J* = 271.5 Hz), 46.73. IR (cm^{-1}): 1601(s), 1532(s), 1483(m), 1362(s), 1313(m), 1235(s), 1166(s), 1117(m), 1036(s), 967(s), 858(s), 742(s). HRMS: calcd for $C_{27}H_{19}F_6N_2O_5S$ [M + H$^+$] 597.0919; found 597.0924.

(3*E*,5*E*)-3,5-bis(2-(trifluoromethyl)benzylidene)-1-((4-(trifluoromethyl)phenyl)sulfonyl)piperidin-4-one (**7**)

Yellow powder, yield: 82%; m.p. 163~165°C. ^{1}H NMR (600 MHz, DMSO) δ 7.89 (d, *J* = 7.9 Hz, 2H), 7.83 – 7.78 (m, 6H), 7.69 (dd, *J* = 12.6, 6.8 Hz, 4H), 7.50 (d, *J* = 7.6 Hz, 2H), 4.54 (s, 4H). ^{13}C NMR (150 MHz, DMSO) δ 183.76, 152.68, 141.90, 133.94, 133.37, 132.81, 132.17, 131.25, 130.47, 129.24 (q, *J* = 31.8 Hz), 128.37, 128.14 (q, *J* = 29.7 Hz), 125.35 (q, *J* = 29.7 Hz), 124.38 (q, *J* = 272.3 Hz), 123.72, 46.67. IR (cm^{-1}): 1674(s), 1613(s), 1483(s), 1451(s), 1403(s), 1359(s), 1320(s), 1217(s), 1168(s), 1061(s), 985(s), 847(s), 759(s). HRMS: calcd for $C_{28}H_{19}F_9NO_3S$ [M + H$^+$] 620.0942; found 620.0940.

(3*E*,5*E*)-1-((4-fluorophenyl)sulfonyl)-3,5-bis(2-(trifluoromethyl)benzylidene)piperidin-4-one (**8**)

Yellow powder, yield: 85%; m.p. 247~249°C. ^{1}H NMR (600 MHz, DMSO) δ 7.90 (d, *J* = 7.9 Hz, 2H), 7.71 (d, *J* = 7.9 Hz, 2H), 7.59 (m, 4H), 7.49 (t, *J* = 7.3 Hz, 2H), 7.41 (t, *J* = 8.0 Hz, 2H), 7.39 (d, *J* = 7.8 Hz, 2H), 4.64 (s, 4H). ^{13}C NMR (150 MHz, DMSO) δ 184.03, 160.75 (d, *J* = 247.5 Hz), 141.98, 133.60 (q, *J* = 32.5 Hz), 132.81 (d, *J* = 8.7 Hz), 132.37, 131.55, 130.37 (d, *J* = 3.7 Hz), 128.58, 127.11 (q, *J* = 3.4 Hz), 125.43 (d, *J* = 2.9 Hz), 123.80 (q, *J* = 271.5 Hz), 122.03 (d, *J* = 13.2 Hz), 116.57 (d, *J* = 21.6 Hz), 47.03. IR (cm^{-1}): 2592(m), 1691(s), 1611(m), 1576(s), 1450(m), 1316(s), 1160(s), 1120(m), 1059(s), 1035(s), 955(m), 825(s), 766(s). HRMS: calcd for $C_{27}H_{19}F_7NO_3S$ [M + H$^+$] 570.0974; found 570.0970.

(3*E*,5*E*)-1-((4-chlorophenyl)sulfonyl)-3,5-bis(2-(trifluoromethyl)benzylidene)piperidin-4-one (**9**)

Yellow powder, yield: 82%; m.p. 182~184°C. ^{1}H NMR (600 MHz, DMSO) δ 7.60 (m, 6H), 7.49 (m, 4H), 7.41 (d, *J* = 8.4 Hz, 2H), 7.38 (d, *J* = 8.4 Hz, 2H), 4.60 (s, 4H). ^{13}C NMR (150 MHz, DMSO) δ 183.79, 139.01, 136.67, 133.89, 133.38, 132.95, 132.23, 131.23, 130.45, 130.11, 129.32, 128.13 (q, *J* = 29.6 Hz), 127.08 (q, *J* = 5.2 Hz), 124.40 (q, *J* = 272.0 Hz), 46.73. IR (cm^{-1}): 2832(m), 1676(s), 1616(s), 1575(m), 1481(s), 1451(s), 1352(s), 1256(s), 1170(s), 1095(s), 1049(s), 993(s), 835(s), 756(s). HRMS: calcd for $C_{27}H_{19}F_6ClNO_3S$ [M + H$^+$] 586.0678; found 586.0683.

(3*E*,5*E*)-1-((4-bromophenyl)sulfonyl)-3,5-bis(2-(trifluoromethyl)benzylidene)piperidin-4-one (**10**)

Yellow powder, yield: 80%; m.p. 197~199°C. ^{1}H NMR (600 MHz, DMSO) δ 7.92 (d, *J* = 7.8 Hz, 2H), 7.83 (m, 4H), 7.72 (d, J = 7.8 Hz, 2H), 7.70 (d, *J* = 8.4 Hz, 2H), 7.52 (d, *J* = 7.6 Hz, 2H), 7.44 (d, *J* = 8.6 Hz, 2H), 4.51 (s, 4H). ^{13}C NMR (150 MHz, DMSO) δ 183.81, 137.03, 133.90, 133.36, 133.05, 132.95, 132.23, 131.22, 130.43, 129.35, 128.13 (q, *J* = 30.2 Hz), 128.06, 127.07 (q, *J* = 5.6 Hz), 124.40 (q, *J* = 271.5 Hz), 46.72. IR (cm^{-1}): 1681(s), 1574(s), 1488(s), 1352(s), 1318(s), 1237(s), 1160(s), 1109(m), 1060(s), 1035(s), 1010(s), 966(s), 840(s), 754(s). HRMS: calcd for $C_{27}H_{19}BrF_6NO_3S$ [M + H$^+$] 630.0173; found 630.0164.

(3*E*,5*E*)-1-((4-cyanophenyl)sulfonyl)-3,5-bis(2-(trifluoromethyl)benzylidene)piperidin-4-one (**11**)

Yellow powder, yield: 78%; m.p. 236~238°C. ^{1}H NMR (600 MHz, DMSO) δ 7.98 (d, *J* = 8.5 Hz, 2H), 7.92 (d, *J* = 7.8 Hz, 2H), 7.86 – 7.81 (m, 4H), 7.71 (dd, *J* = 12.3, 8.1 Hz, 4H), 7.52 (d, *J* = 7.7 Hz, 2H), 4.55 (s, 4H). ^{13}C NMR (150 MHz, DMSO) δ 183.82, 142.09, 134.09, 133.99, 133.39, 132.82, 132.16, 131.24, 130.48, 128.14 (d, *J* = 29.8 Hz), 128.13, 127.08 (q, *J* = 4.5 Hz), 126.88, 124.40 (q, *J* = 271.5 Hz), 46.72. IR (cm^{-1}): 2229(s), 1681(s), 1618(s), 1488(s), 1356(s), 1317(s), 1163(s), 1114(s), 1060(s), 1035(s), 965(s), 850(m), 771(s). HRMS: calcd for $C_{27}H_{19}F_6N_2O_3S$ [M + H$^+$] 565.1021; found 565.1014.

(3*E*,5*E*)-1-((4-acetamidophenyl)sulfonyl)-3,5-bis(2-(trifluoromethyl)benzylidene)piperidin-4-one (**12**)

Yellow powder, yield: 76%; m.p. 238~240°C. ^{1}H NMR (600 MHz, DMSO) δ 10.40 (s, 1H), 7.92 (d, *J* = 7.8 Hz, 2H), 7.84 (t, *J* = 7.8 Hz, 2H), 7.81 (s, 2H), 7.71 (m, 4H), 7.51 (d, *J* = 7.2 Hz, 2H), 7.41

(d, J = 8.4 Hz, 2H), 4.50 (s, 4H), 2.11 (s, 3H). ^{13}C NMR (150 MHz, DMSO) δ 182.63, 168.57, 143.20, 132.64, 132.30, 132.11, 131.28, 130.22, 129.97, 129.33, 127.67, 127.08 (q, J = 29.7 Hz), 126.04 (q, J = 4.5 Hz), 125.51, 123.36 (q, J = 273.0 Hz), 45.79, 23.62. IR (cm^{-1}): 3371(m), 2838(s), 1706(s), 1621(s), 1591(s), 1401(s), 1340(s), 1314(s), 1284(s), 1231(s), 1151(s), 1134(s), 1060(s), 1038(s), 957(s), 857(s), 765(s). HRMS: calcd for $C_{29}H_{23}F_6N_2O_4S$ [M + H$^+$] 609.1283; found 609.1280.

(3*E*,5*E*)-1-(phenylsulfonyl)-3,5-bis(3-(trifluoromethyl)benzylidene)piperidin-4-one (**13**)

Yellow powder, yield: 88%; m.p. 151~153℃. ^{1}H NMR (600 MHz, DMSO) δ 7.86 (d, J = 6.7 Hz, 2H), 7.84 (s, 2H), 7.80 (m, 4H), 7.73 (t, J = 7.2 Hz, 1H), 7.67 (s, 2H), 7.56 (t, J = 7.4 Hz, 2H), 7.53 (d, J = 7.1 Hz, 2H), 4.63 (s, 4H). ^{13}C NMR (150 MHz, DMSO) δ 184.48, 137.49, 136.18, 135.46, 134.35, 134.20, 132.50, 130.54, 130.10 (q, J = 31.8 Hz), 129.96, 127.60, 127.40 (q, J = 3.5 Hz), 126.55 (q, J = 3.5 Hz), 124.43 (q, J = 271.5 Hz), 46.96. IR (cm^{-1}): 1678(s), 1592(s), 1486(s), 1344(s), 1286(s), 1242(s), 1169(s), 1116(m), 1086(s), 1056(s), 1037(s), 990(s), 810(m), 734(s). HRMS: calcd for $C_{27}H_{20}F_6NO_3S$ [M + H$^+$] 552.1068; found 552.1070.

(3*E*,5*E*)-1-tosyl-3,5-bis(3-(trifluoromethyl)benzylidene)piperidin-4-one (**14**)

Yellow powder, yield: 82%; m.p. 155~158℃. ^{1}H NMR (600 MHz, DMSO) δ 7.85 (d, J = 8.6 Hz, 2H), 7.84 (s, 2H), 7.79 (m, 4H), 7.69 (s, 2H), 7.42 (d, J = 8.4 Hz, 2H), 7.36 (d, J = 8.4 Hz, 2H), 4.59 (s, 4H), 2.41 (s, 3H). ^{13}C NMR (150 MHz, DMSO) δ 184.57, 144.67, 136.12, 135.48, 134.42, 134.34, 132.60, 130.53, 130.39, 130.08 (q, J = 31.8 Hz), 127.71, 127.39 (q, J = 3.4 Hz), 126.53 (q, J = 3.5 Hz), 124.43 (q, J =270.4 Hz), 46.97, 21.51. IR (cm^{-1}): 2930(m), 1677(s), 1510(s), 1430(s), 1345(s), 1320(s), 1287(s), 1164(s), 1118(s), 1014(s), 988(s), 921(s), 843(s), 754(s). HRMS: calcd for $C_{28}H_{22}F_6NO_3S$ [M + H$^+$] 566.1225; found 566.1219.

(3*E*,5*E*)-1-((4-nitrophenyl)sulfonyl)-3,5-bis(3-(trifluoromethyl)benzylidene)piperidin-4-one (**15**)

Yellow powder, yield: 85%; m.p. 196~198℃. ^{1}H NMR (600 MHz, DMSO) δ 8.34 (d, J = 8.8 Hz, 2H), 7.89 (s, 2H), 7.86 (d, J = 7.2 Hz, 2H), 7.80 (m, 6H), 7.67 (s, 2H), 4.74 (s, 4H). ^{13}C NMR (150 MHz, DMSO) δ 184.55, 150.57, 143.47, 136.60, 135.36, 134.30, 132.11, 130.53, 130.15 (q, J = 31.9 Hz), 129.28, 127.68 (q, J = 3.8 Hz), 126.65 (q, J = 3.5 Hz), 125.33, 124.43 (q, J = 271.5 Hz),

46.93. IR (cm^{-1}): 2818(s), 1676(s), 1589(s), 1433(s), 1350(s), 1317(s), 1278(s), 1168(s), 1118(s), 1033(s), 957(s), 845(s), 736(s). HRMS: calcd for $C_{27}H_{19}F_6N_2O_5S$ [M + H^+] 597.0919; found 597.0920.

(3*E*,5*E*)-3,5-bis(3-(trifluoromethyl)benzylidene)-1-((4-(trifluoromethyl)phenyl)sulfonyl)piperidin-4-one (**16**)

Yellow powder, yield: 81%; m.p. 148~150°C. ^{1}H NMR (600 MHz, DMSO) δ 7.93 (d, *J* = 8.3 Hz, 2H), 7.86 (d, *J* = 9.5 Hz, 4H), 7.78 (m, 6H), 7.68 (s, 2H), 4.70 (s, 4H). ^{13}C NMR (150 MHz, DMSO) δ 184.51, 141.67, 136.37, 135.34, 134.29, 133.59 (q, *J* = 32.6 Hz), 132.28, 130.55, 130.13 (q, *J* = 31.8 Hz), 128.69, 127.57 (q, *J* = 3.4 Hz), 127.15 (q, *J* = 3.4 Hz), 126.64 (q, *J* = 3.5 Hz), 124.42 (q, *J* =271.1 Hz), 123.80 (q, *J* = 271.8 Hz), 46.89. IR (cm^{-1}): 2819(s), 1930(m), 1676(s), 1608(s), 1420(s), 1352(s), 1317(s), 1274(s), 1166(s), 1116(s), 1027(s), 993(s), 850(s), 724(s). HRMS: calcd for $C_{28}H_{19}F_9NO_3S$ [M + H^+] 620.0942; found 620.0931.

(3*E*,5*E*)-1-((4-fluorophenyl)sulfonyl)-3,5-bis(3-(trifluoromethyl)benzylidene)piperidin-4-one (**17**)

Yellow powder, yield: 87%; m.p. 175~177°C. ^{1}H NMR (600 MHz, DMSO) δ 7.86 (s, 2H), 7.85 (d, *J* = 7.2 Hz, 2H), 7.79 (dt, *J* = 15.5, 7.8 Hz, 4H), 7.68 (s, 2H), 7.60 (dd, *J* = 8.9, 5.1 Hz, 2H), 7.40 (t, *J* = 8.7 Hz, 2H), 4.67 (s, 4H). ^{13}C NMR (150 MHz, DMSO) δ 183.41, 164.24 (d, *J* = 252.8 Hz), 135.28, 134.37, 133.29, 133.02 (d, *J* = 3.0 Hz), 131.32, 129.78 (d, *J* = 9.8 Hz), 129.48, 129.07 (q, *J* = 31.9 Hz), 126.44 (d, *J* = 3.9 Hz), 125.53 (d, *J* = 3.4 Hz), 123.37 (q, *J* = 270.0 Hz), 116.14 (d, *J* = 22.8 Hz), 45.91. IR (cm^{-1}): 3095(s), 1676(s), 1594(s), 1488(s), 1353(s), 1315(s), 1277(s), 1162(s), 1116(s), 1021(s), 988(s), 958(s), 843(s), 722(s). HRMS: calcd for $C_{27}H_{19}F_7NO_3S$ [M + H^+] 570.0974; found 570.0965.

(3*E*,5*E*)-1-((4-chlorophenyl)sulfonyl)-3,5-bis(3-(trifluoromethyl)benzylidene)piperidin-4-one (**18**)

Yellow powder, yield: 85%; m.p. 159~161°C. ^{1}H NMR (600 MHz, DMSO) δ 7.86 (s, 2H), 7.85 (d, *J* = 7.4 Hz, 2H), 7.79 (dt, *J* = 15.3, 7.8 Hz, 4H), 7.69 (s, 2H), 7.63 (d, *J* = 8.7 Hz, 2H), 7.54 (d, *J* = 8.7 Hz, 2H), 4.67 (s, 4H). ^{13}C NMR (150 MHz, DMSO) δ 184.50, 139.11, 136.52, 136.36, 135.42, 134.32, 132.34, 130.54, 130.12 (q, *J* = 31.8 Hz), 130.10, 129.59, 127.53 (q, *J* = 3.5 Hz), 126.59 (q, *J* = 3.5 Hz), 124.42 (q, *J* = 270.0 Hz), 46.95. IR (cm^{-1}): 2818(s), 1676(s), 1577(s), 1483(s), 1352(s),

1315(s), 1289(s), 1160(s), 1118(s), 1027(s), 988(s), 843(s), 712(s). HRMS: calcd for $C_{27}H_{19}F_6ClNO_3S$ [M + H$^+$] 586.0678; found 586.0672.

(3*E*,5*E*)-1-((4-bromophenyl)sulfonyl)-3,5-bis(3-(trifluoromethyl)benzylidene)piperidin-4-one (**19**)
Yellow powder, yield: 82%; m.p. 135~137°C. ^{1}H NMR (600 MHz, DMSO) δ 7.86 (s, 2H), 7.85 (d, *J* = 7.0 Hz, 2H), 7.80 (t, *J* = 6.4 Hz, 3H), 7.77 (d, *J* = 8.5 Hz, 3H), 7.69 (s, 2H), 7.47 (d, *J* = 8.6 Hz, 2H), 4.66 (s, 4H). ^{13}C NMR (150 MHz, DMSO) δ 183.46, 135.84, 135.29, 134.34, 133.26, 131.99, 131.29, 129.48, 129.07 (q, *J* = 31.8 Hz), 128.56, 127.12, 126.48 (q, *J* = 3.5 Hz), 125.54 (q, *J* = 3.4 Hz), 123.37 (q, *J* = 271.5 Hz), 45.89. IR (cm^{-1}): 3094(s), 2818(s), 1675(s), 1610(s), 1475(s), 1360(s), 1320(s), 1265(s), 1165(s), 1115(s), 1024(s), 988(s), 825(s), 737(s). HRMS: calcd for $C_{27}H_{19}BrF_6NO_3S$ [M + H$^+$] 630.0173; found 630.0170.

(3*E*,5*E*)-1-((4-cyanophenyl)sulfonyl)-3,5-bis(3-(trifluoromethyl)benzylidene)piperidin-4-one (**20**)
Yellow powder, yield: 83%; m.p. 157~160°C. ^{1}H NMR (600 MHz, DMSO) δ 8.05 (d, *J* = 8.5 Hz, 2H), 7.89 (s, 2H), 7.86 (d, *J* = 7.6 Hz, 2H), 7.82 (d, *J* = 7.8 Hz, 2H), 7.79 (t, *J* = 7.6 Hz, 2H), 7.69 (d, *J* = 8.5 Hz, 2H), 7.66 (s, 2H), 4.73 (s, 4H). ^{13}C NMR (150 MHz, DMSO) δ 184.49, 141.98, 136.53, 135.35, 134.35, 134.06, 132.11, 130.54, 130.15 (q, *J* = 31.8 Hz), 128.38, 127.60 (q, *J* = 3.5 Hz), 126.64 (q, *J* = 3.3 Hz), 124.42 (q, *J* = 270.0 Hz), 118.0, 116.4, 46.93. IR (cm^{-1}): 3095(s), 2818(s), 1676(s), 1594(s), 1488(s), 1352(s), 1315(s), 1289(s), 1160(s), 1118(s), 1027(s), 992(s), 958(s), 839(s), 737(s). HRMS: calcd for $C_{27}H_{19}F_6N_2O_3S$ [M + H$^+$] 565.1021; found 565.1017.

(3*E*,5*E*)-1-((4-acetamidophenyl)sulfonyl)-3,5-bis(3-(trifluoromethyl)benzylidene)piperidin-4-one (**21**)
Yellow powder, yield: 87%; m.p. 202~204°C. ^{1}H NMR (600 MHz, DMSO) δ 10.41 (s, 1H), 7.86 (d, *J* = 6.2 Hz, 2H), 7.84 (s, 2H), 7.79 (m, 3H), 7.76 (d, *J* = 8.8 Hz, 2H), 7.70 (s, 2H), 7.48 (d, *J* = 8.8 Hz, 2H), 4.57 (s, 4H), 2.12 (s, 3H). ^{13}C NMR (150 MHz, DMSO) δ 184.51, 169.65, 144.35, 136.11, 135.48, 134.35, 132.65, 130.54, 130.43, 130.10 (q, *J* = 32.9 Hz), 129.00, 127.39 (q, *J* = 3.8 Hz), 126.53 (q, *J* = 3.6 Hz), 124.42 (q, *J* = 271.5 Hz), 119.07, 47.09, 24.67. IR (cm^{-1}): 3324(s), 2818(s), 1684(s), 1592(s), 1468(s), 1344(s), 1320(s), 1275(s), 1164(s), 1123(s), 1027(s), 957(m), 842(s), 742(s). HRMS: calcd for $C_{29}H_{23}F_6N_2O_4S$ [M + H$^+$] 609.1283; found 609.1282.

(3*E*,5*E*)-1-(phenylsulfonyl)-3,5-bis(4-(trifluoromethyl)benzylidene)piperidin-4-one (**22**)

Yellow powder, yield: 90%; m.p. 234~236°C. ^{1}H NMR (400 MHz, DMSO) δ 7.89 (d, *J* = 8.2 Hz, 4H), 7.71 (d, *J* = 8.2 Hz, 4H), 7.70 (m, 1H), 7.62 (s, 2H), 7.57 (t, *J* = 8.2 Hz, 2H), 7.52 (d, *J* = 7.1 Hz, 2H), 4.68 (s, 4H). ^{13}C NMR (100 MHz, DMSO) δ 184.67, 138.64, 138.00, 136.37, 134.37, 133.10, 131.71, 130.21, 130.10 (q, *J* = 30.0 Hz), 127.90, 126.42 (q, *J* = 3.7 Hz), 124.72 (q, *J* = 270.0 Hz), 47.23. IR (cm^{-1}): 3068(m), 1679(s), 1619(s), 1484(s), 1433(m), 1331(s), 1284(s), 1245(s), 1189(s), 1168(s), 1118(s), 1074(s), 1040(s), 993(s), 801(s), 727(s). HRMS: calcd for $C_{27}H_{20}F_6NO_3S$ [M + H$^+$] 552.1068; found 552.1064.

(3*E*,5*E*)-3,5-bis(4-(trifluoromethyl)benzylidene)-1-((4-(trifluoromethyl)phenyl)sulfonyl)piperidin-4-one (**25**)

Yellow powder, yield: 81%; m.p. 224~226°C. ^{1}H NMR (400 MHz, DMSO) δ 7.96 (d, *J* = 8.3 Hz, 2H), 7.89 (d, *J* = 8.3 Hz, 4H), 7.79 (d, *J* = 8.2 Hz, 2H), 7.72 (d, *J* = 8.2 Hz, 4H), 7.65 (s, 2H), 4.71 (s, 4H). ^{13}C NMR (100 MHz, DMSO) δ 184.71, 141.92, 138.52, 136.56, 133.82 (q, *J* = 31.8 Hz), 132.89, 131.77, 130.12 (q, *J* = 32.1 Hz), 129.02, 127.43 (q, *J* = 3.5 Hz), 126.43 (q, *J* = 3.7 Hz), 124.72 (q, *J* = 270.7 Hz), 124.05 (q, *J* = 270.2 Hz), 47.25. IR (cm^{-1}):2824(s), 1932(m), 1676(s), 1616(s), 1583(s), 1413(s), 1371(s), 1320(s), 1265(s), 1238(s), 1172(s), 1113(s), 1070(s), 994(s), 860(s), 713(s). HRMS: calcd for $C_{28}H_{19}F_9NO_3S$ [M + H$^+$] 620.0942; found 620.0935.

(3*E*,5*E*)-1-((4-chlorophenyl)sulfonyl)-3,5-bis(4-(trifluoromethyl)benzylidene)piperidin-4-one (**27**)

Yellow powder, yield: 76%; m.p. 220~222°C. ^{1}H NMR (400 MHz, DMSO) δ 7.89 (d, *J* = 8.3 Hz, 4H), 7.72 (d, *J* = 8.2 Hz, 4H), 7.65 (d, *J* = 8.4 Hz, 2H), 7.64 (s, 2H), 7.54 (d, *J* = 8.7 Hz, 2H), 4.69 (s, 4H). ^{13}C NMR (100 MHz, DMSO) δ 184.71, 139.33, 138.60, 136.88, 136.56, 132.96, 131.76, 130.34, 130.09 (q, *J* = 32.3 Hz), 129.90, 126.42 (q, *J* = 3.7 Hz), 124.72 (q, *J* = 270.2 Hz), 47.26. IR (cm^{-1}): 3094(s), 2822(s), 1674(s), 1614(s), 1583(s), 1478(s), 1414(s), 1367(s), 1321(s), 1265(s), 1171(s), 1114(s), 1069(s), 969(s), 826(s), 756(s). HRMS: calcd for $C_{27}H_{19}F_6ClNO_3S$ [M + H$^+$] 586.0678; found 586.0669.

(3*E*,5*E*)-1-((4-bromophenyl)sulfonyl)-3,5-bis(4-(trifluoromethyl)benzylidene)piperidin-4-one (**28**)

Yellow powder, yield: 83%; m.p. 218~220°C. ^{1}H NMR (400 MHz, DMSO) δ 7.89 (d, *J* = 8.3 Hz, 4H), 7.79 (d, *J* = 8.6 Hz, 2H), 7.72 (d, *J* = 8.2 Hz, 4H), 7.65 (s, 2H), 7.47 (d, *J* = 8.6 Hz, 2H), 4.68 (s, 4H). ^{13}C NMR (100 MHz, DMSO) δ 184.73, 138.58, 137.24, 136.55, 133.28, 132.97, 131.76, 130.10 (q, *J* = 31.0 Hz), 129.94, 128.42, 126.42 (q, *J* = 3.7 Hz), 124.42 (q, *J* = 270.4 Hz), 47.26. IR (cm^{-1}): 3094(s), 2822(s), 1674(s), 1573(s), 1474(s), 1413(s), 1368(s), 1320(s), 1264(s), 1235(s), 1171(s), 1115(s), 1068(s), 937(s), 825(s), 737(s). HRMS: calcd for $C_{27}H_{19}BrF_6NO_3S$ [M + H^+] 630.0173; found 630.0168.

(3*E*,5*E*)-1-((4-acetamidophenyl)sulfonyl)-3,5-bis(4-(trifluoromethyl)benzylidene)piperidin-4-one (**30**)

Yellow powder, yield: 86%; m.p. 233~235°C. ^{1}H NMR (400 MHz, DMSO) δ 10.39 (s, 1H), 7.89 (d, *J* = 8.2 Hz, 4H), 7.74 (d, *J* = 8.8 Hz, 2H), 7.70 (d, *J* = 8.1 Hz, 4H), 7.65 (s, 2H), 7.50 (d, *J* = 8.8 Hz, 2H), 4.61 (s, 4H), 2.12 (s, 3H). ^{13}C NMR (100 MHz, DMSO) δ 184.00, 169.16, 143.75, 137.93, 135.54, 132.54, 130.98, 130.22, 129.30 (q, *J* = 31.9 Hz), 128.60, 125.68 (q, *J* = 3.7 Hz), 123.98 (q, *J* = 271.0 Hz), 118.63, 46.57, 24.19. IR (cm^{-1}): 3326(m), 2822(s), 1692(s), 1615(s), 1587(s), 1530(s), 1322(s), 1262(s), 1169(s), 1128(s), 1069(s), 1015(s), 956(m), 845(s), 771(s). HRMS: calcd for $C_{29}H_{23}F_6N_2O_4S$ [M + H^+] 609.1283; found 609.1276.

Further in vitro anti-tumor activity testing of BAPs (4-30) with MTT method

To further verify the anti-tumor activity, one human non-small cell lung cancer cell line (A549), one human ovarian cancer cell line (A2780), and one human normal heptical cell line (HHL-5) were screened by BAPs (**4-30**) using a modified MTT assay (Dojindo Laboratories, Tokyo, Japan). The A549, A2780, and HHL-5 cell lines were maintained at 37°C, 5% humidified CO_2, and 95% atmosphere. DMEM medium containing 10% FBS was used for culturing the A549 and A2780 cell lines, meanwhile, RPMI 1640 medium containing 10% FBS was used for culturing the HHL-5 cell line. The A549, A2780, and HHL-5 cells were seeded in a 96-well plate in 200 μL of medium per well at a density of approximately 8×10^3 cells/well, and cultured for 24 hours, followed by incubation in an incubator of indicated compounds with successive concentrations (10, 5, 2.5, 1.25, 0.625, 0.3125 μg/mL) for 24 h. In the control group, cells were only cultured with culture media. After the media was removed, 20 μL of MTT (5 mg/mL) was added, then cells were incubated for 4

h at 37℃. Next, removed the media with MTT and added 150 μL of DMSO to dissolve the dark-blue formazan crystals. The optical density (OD) value of each well was measured on a multi-well plate reader (TECAN, Männedorf, Switzerland) at 570 nm. GraphPad Prism 5 software was used to calculate their IC_{50} values. Every IC_{50} value was the average of three replicates. Doxorubicin (DOX) and Curcumin were used as positive controls. The concentrations of DOX used were 5, 2.5, 1.25, 0.625, 0.3125, 0.15625 μg/mL. The concentrations of Curcumin used were 100, 50, 25, 12.5, 6.25, 3.125, 1, 0.5 and 0.1 μg/mL. Results are the average of three replicates and shown in Table S1.

Table S1. Cytotoxicity of BAPs (**4-30**), Curcumin, and DOX.

Compound	R_1	R_2	A549 (μM)	SI[a]	A2780 (μM)	SI[a]	HHL-5 (μM)
**4**	2-CF_3	-H	2.24 ± 1.23	3.8	54.04 ± 11.99	0.2	8.6 ± 0. 8
**5**	2-CF_3	-CH_3	13.97 ± 1.99	1.8	4.85±0.47	5.1	24.9 ± 0.1
**6**	2-CF_3	-NO_3	7.58 ± 1.76	1.0	28.8 ± 5.33	0.3	7.4 ± 0. 7
**7**	2-CF_3	-CF_3	2.68 ± 0.53	3.4	0.33 ± 0.03	27.6	9.1 ± 0.2
**8**	2-CF_3	-F	13.45 ± 1.39	0.9	1.44 ± 0.54	8.2	11.8 ± 0.2
**9**	2-CF_3	-Cl	1.86 ± 0.61	5.0	0.62 ± 0.07	15.0	9.3 ± 0.1
**10**	2-CF_3	-Br	5.01 ± 0.45	2.9	2.34 ± 0.39	6.2	14.5 ± 0.1
**11**	2-CF_3	-CN	64.19 ± 10.17	0.2	32.3 ± 7.69	0.5	15.5 ± 0.3
**12**	2-CF_3	-NHAc	2.39 ± 0.09	3.3	1.22 ± 0.06	6.5	7.9 ± 0.2
**13**	3-CF_3	-H	0.25 ± 0.01	11.6	0.16 ± 0.01	18.1	2.9 ± 0.2
**14**	3-CF_3	-CH_3	0.23 ± 0.04	40.9	0.14 ± 0.03	67.1	9.4 ± 0.2
**15**	3-CF_3	-NO_3	0.13 ± 0.02	40.0	0.14 ± 0.02	37.1	5.2 ± 0.3
**16**	3-CF_3	-CF_3	0.43 ± 0.08	14.4	0.27 ± 0.02	23.0	6.2 ± 0.4
**17**	3-CF_3	-F	0.28 ± 0.01	12.5	0.14 ± 0.01	25.0	3.5 ± 0.5
**18**	3-CF_3	-Cl	0.15 ± 0.02	14.7	0.19 ± 0.02	11.6	2.2 ± 0.1
**19**	3-CF_3	-Br	0.12 ± 0.01	113.3	0.21 ± 0.03	64.8	13.6±0.5
**20**	3-CF_3	-CN	0.17 ± 0.002	14.1	0.15 ± 0.02	16.0	2.4±0.4
**21**	3-CF_3	-NHAc	0.21 ± 0.01	14.3	0.17 ± 0.01	17.6	3.0 ± 0.6
**22**	4-CF_3	-H	0.18 ± 0.02	43.9	0.17 ± 0.06	46.5	7.9 ± 0.1
**23**	4-CF_3	-CH_3	0.15 ± 0.02	23.3	0.16 ± 0.02	21.9	3.5 ± 0.2
**24**	4-CF_3	-NO_3	0.17 ± 0.01	44.1	0.17 ± 0.02	44.1	7.5 ± 0.6
**25**	4-CF_3	-CF_3	0.27 ± 0.03	41.1	0.28 ± 0.03	39.6	11.1 ± 0.2
**26**	4-CF_3	-F	0.16 ± 0.05	70.6	0.297 ± 0.05	38.0	11. 3 ± 0.1
**27**	4-CF_3	-Cl	0.14 ± 0.04	65.0	0.197 ± 0.05	46.2	9.1 ± 0.2
**28**	4-CF_3	-Br	0.20 ± 0.02	103.5	0.18 ± 0.04	115.0	20.7 ± 0.5
**29**	4-CF_3	-CN	0.081 ± 0.004	203.7	0.18 ± 0.01	91.7	16.5 ± 0.3
**30**	4-CF_3	-NHAc	0.098 ± 0.01	21.4	0.13 ± 0.02	16.2	2.1 ± 0.1
DOX	-	-	0.29 ± 0.07	42.1	0.19 ± 0.01	64.2	12.2 ± 0.4
Curcumin	-	-	32.04 ± 3.55	0.8	44.04 ± 9.82	0.5	24.2 ± 0.5

[a]The letters SI refer to the selectivity index which is the quotient of the IC_{50} values for normal and malignant cells.

Preliminary safety evaluation of BAP 16

Toxicity test on normal non-liver cell lines: To validate the safety of BAP **16**, one Human embryonic lung fibroblasts cell line (HFL1), one human embryonic kidney cell line (HEK293), and one human normal lung bronchial epithelial cell line (BEAS-2B) were tested using a modified MTT assay (Dojindo Laboratories, Tokyo, Japan). F12K medium containing 10% FBS was used for culturing the HFL1 cell line, DMEM medium containing 10% FBS and 1.5 g/L sodium bicarbonate was used for culturing the HEK293 cell line, and the BEAS-2B special medium was used for culturing BEAS-2B cell line. The remaining operations were consistent with the previous description. Results are the average of three replicates and shown in Table S2.

From the above experimental results, BAP **16** showed moderate cytotoxicity to this three human normal non-heptical cell lines (HFL1, HEK293 and BEAS-2B), which was similar to that of human normal heptical cell lines.

Table S2. Toxicity test of BAP **16** on normal non-heptical cell lines.

Compound	R_1	R_2	HFL1 (μM)	HEK293 (μM)	BEAS-2B (μM)
**16**	3-CF_3	-CF_3	9.62 ± 1.52	7.19 ± 0.91	17.58 ± 2.53

Acute Toxicity Studies: Animal experiments were reviewed and approved by the Binzhou Medical University Experimental Animal Committee. The acute toxicity study was investigated on the six-week-old female ICR mice (n = 30) weighing 18-22 grams (Jinan Pengyue Experimental Animal Breeding Co., Ltd.). Briefly, mice were randomly divided into three groups (n = 10/group) and administrated the BAP **16** or solvent. The three groups of mice were treated with intragastric administration of 300 mg/kg, 600 mg/kg of BAP **16** and vehicle (saline containing 0.5% CMC-Na) each day. The body weight of mice was recorded every day, and the toxicity and death of mice were monitored daily for up to day 14. On day 14, the surviving animals were dissected to observe whether the internal organs had visible tissue changes, and then the livers were taken for formaldehyde fixation, dehydration, embedding, sectioning, and HE staining. The cell images were taken using a light microscopy (Olympus, Tokyo, Japan).

During the 14 days of intragastric administration, the food and water intakes of all three groups of mice were normal, the body weight increased slightly, and no obvious toxic reaction was observed. However, in the HE staining experiment, some tissues of mouse livers showed dose-related toxic changes, such as smaller hepatocyte volume, deeper nuclear staining, eosinophilic changes, and edema (Figure S1).

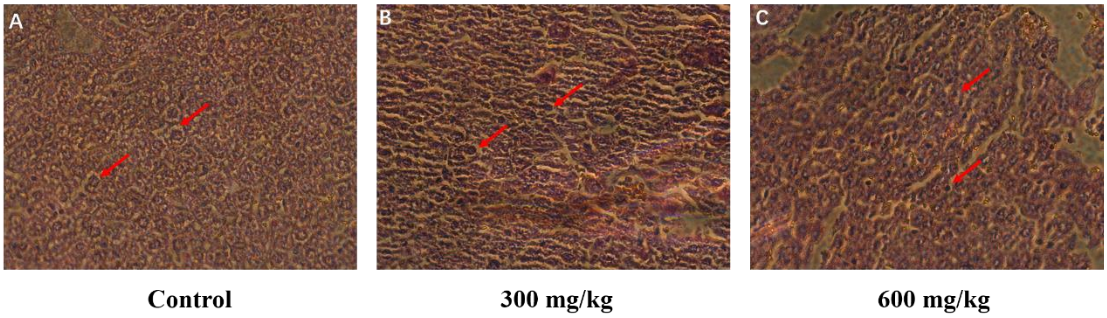

Figure S1. The morphology of the liver tissues after the photo by HE staining. (A) In the control group, the hepatic lobular cells were normal without edema and degeneration. (B) In the 300 mg/kg group, it was observed that some liver cells became smaller, the cytoplasm was red stained, and the nucleus stained deeper. It showed eosinophilic changes, and there was some liver tissue edema. (C) In the 600 mg/kg group, the cytoplasm of mouse liver was dehydrated seriously, the volume became smaller and the color of nucleus became deeper. There were obvious eosinophilic changes and edema.
